# Distinctive Supramolecular Features of β-Cyclodextrin Inclusion Complexes with Antidepressants Protriptyline and Maprotiline: A Comprehensive Structural Investigation

**DOI:** 10.3390/ph14080812

**Published:** 2021-08-18

**Authors:** Thammarat Aree

**Affiliations:** Department of Chemistry, Faculty of Science, Chulalongkorn University, Bangkok 10330, Thailand; athammar@chula.ac.th; Tel.: +66-2-2187584; Fax: +66-2-2187598

**Keywords:** β-cyclodextrin, protriptyline, maprotiline, tricyclic antidepressants (TCAs), X-ray analysis, DFT calculation

## Abstract

Depression, a global mental illness, is worsened due to the coronavirus disease 2019 (COVID-2019) pandemic. Tricyclic antidepressants (TCAs) are efficacious for the treatment of depression, even though they have more side effects. Cyclodextrins (CDs) are powerful encapsulating agents for improving molecular stability, water solubility, and lessening the undesired effects of drugs. Because the atomic-level understanding of the β-CD–TCA inclusion complexes remains elusive, we carried out a comprehensive structural study via single-crystal X-ray diffraction and density functional theory (DFT) full-geometry optimization. Here, we focus on two complexes lining on the opposite side of the β-CD–TCA stability spectrum based on binding constants (*K*_a_s) in solution, β-CD–protriptyline (PRT) **1**—most stable and β-CD–maprotiline (MPL) **2**—least stable. X-ray crystallography unveiled that in the β-CD cavity, the PRT B-ring and MPL A-ring are aligned at a nearly perfect right angle against the O4 plane and primarily maintained in position by intermolecular C–H···π interactions. The increased rigidity of the tricyclic cores is arising from the PRT -CH=CH- bridge widens, and the MPL -CH_2_–CH_2_- flexure narrows the butterfly angles, facilitating the deepest and shallower insertions of PRT B-ring (**1**) and MPL A-ring (**2**) in the distorted round β-CD cavity for better complexation. This is indicated by the DFT-derived complex stabilization energies (Δ*E*_stb_s), although the complex stability orders based on *K*_a_s and Δ*E*_stb_s are different. The dispersion and the basis set superposition error (BSSE) corrections were considered to improve the DFT results. Plus, the distinctive 3D arrangements of **1** and **2** are discussed. This work provides the first crystallographic evidence of PRT and MPL stabilized in the β-CD cavity, suggesting the potential application of CDs for efficient drug delivery.

## 1. Introduction

Depression is a serious mental illness as over 300 million people worldwide suffer from depression, and about 800,000 people die from suicide each year [1]. Due to the emerging coronavirus disease 2019 (COVID-19), a critical question arises: to what extent does the COVID-19 pandemic worsen depression? Recent studies unveiled the alarming statistics: (i) 1.5-year since the COVID-19 outbreak, the number of confirmed COVID-19 cases has topped 170 million across the globe [2]; (ii) 23% of COVID-19 patients worldwide have depression symptoms in common [3]; and (iii) 50% of the survivors suffer from depression [4]. Therefore, both the COVID-19 and its mental effect (depression) require effective treatments concurrently.

**Scheme 1:**
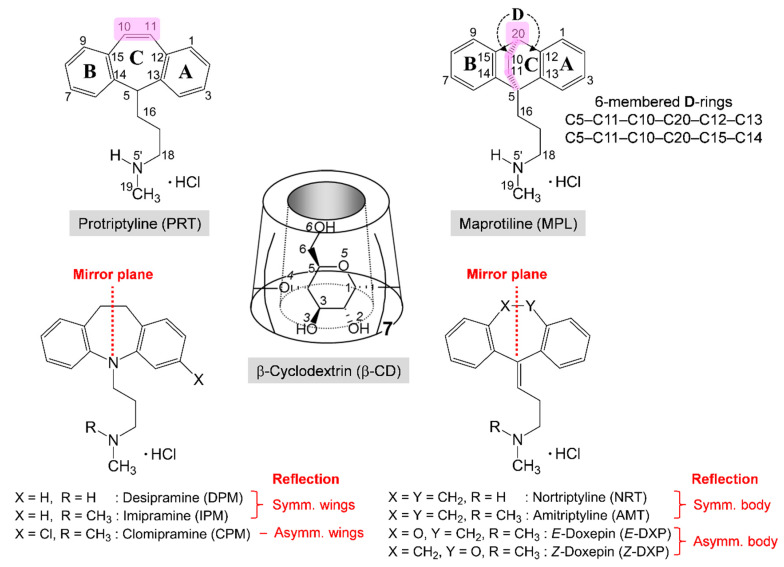
Chemical structures and atom numbering schemes of tricyclic antidepressants (TCAs; butterfly structure) and b-cyclodextrin (β-CD; conical shape). The six widely used TCAs are the secondary amine DPM, NRT, and the tertiary amine IPM, AMT, CPM, *E*/*Z*-DXP. The different TCAs, including PRT and MPL, of which their distinct structure portions are magenta highlighted. TCAs are commercially available in HCl salt form. Note the reflection symmetry of the (6-7-6)-tricyclic core (wing-body-wing) through a vertical mirror plane bisecting the central 7-membered C-ring. Like other TCAs with a skeleton of the B-C-A-rings from left to right, the flexure and the D-rings are beneath the C-ring for the bicyclo[2.2.2]octadiene backbone of MPL.

Tricyclic antidepressants (TCAs) are efficient second-line medications for the treatment of depression. The widely used TCAs include the secondary (2°) amine desipramine (DPM), nortriptyline (NRT), and the tertiary (3°) amine imipramine (IPM), amitriptyline (AMT), clomipramine (CPM), doxepin (DXP), Scheme 1. Although the 2° and 3° amine TCAs share similar structures, they interact differently with neurotransmitters, thus having distinct pharmacological effects. While the 2° amine TCAs selectively inhibit norepinephrine, the 3° amine TCAs block the reuptake of both norepinephrine and serotonin [5]. Protriptyline (PRT; Vivactil) and maprotiline (MPL; Ludiomil) are members of the 2° amine TCAs, thus favoring norepinephrine over serotonin. Like all other TCAs, PRT and MPL mostly share a common structural feature of a butterfly with the aromatic A and B rings as wings and the 3-C-length side-chain as a tail. Their distinct parts are the central C-ring (Scheme 1). MPL with a rigid flexure arising from an ethylene bridge across the central 6-membered C-ring is also categorized as an atypical, tetracyclic antidepressant (TeCA) and is prescribed as the most selective noradrenaline reuptake inhibitor (SNRI) [6]. PRT is a classic TCA with the central cycloheptatriene ring (Scheme 1). Whereas MPL can cause drowsiness and dehydration, PRT can cause heart rhythm problems and affect sex drive [7]. Among five TCAs (PRT, AMT, MPL, DXP, and NRT) exhibiting inhibitory activity against major Alzheimer’s disease (AD) targets, PRT is the most potent multi-target directed ligand for AD treatment [8], which has been clinically tested in rats [9]. Very recent research indicates that MPL has anticancer activity against hepatocellular carcinoma cells [10].

Cyclodextrins (CDs) are the most versatile encapsulating agents because they have a broad application spectrum, including pharmaceutics, cosmetics, medicine, food, agriculture, chromatography, biotechnology, and nanotechnology [11,12,13,14]. The excellent inclusion ability of CDs is attributed to their amphipathic character and nanocavity size, stabilizing and interacting intermolecularly with various guest molecules [15,16]. CDs adopt a shape of hollow, truncated cone with a height of ~8 Å (distance of two hydrophilic rims; wider O2–H/O3–H and narrower O6–H sides) and cavity diameters of ~5–8 Å as they are composed of 6, 7 and 8 D-glucose units connected via α-1,4 glycosidic linkages for α-, β- and γ-CDs, respectively. In pharmaceutical technology, CDs act as water solubilizers and molecular stabilizers against air, light, and heat, improving physicochemical and pharmacological properties and bioavailability of drugs, particularly antidepressants [17]. We have made an insightful literature review on the studies of the CD–TCA inclusion complexes over the past three decades (for 2° amine TCAs in Table 1 and 3° amine TCAs in Table S1), of which their two characteristics of inclusion complexation are summarized as follows.

(i) In solution, the alkylamine side-chain is more favorably entrapped in the CD cavity over the aromatic A/B-ring of the (6-7-6)-tricyclic core, yielding the fairy and moderately highly stable equimolar α-CD and β-CD complexes, respectively. The corresponding binding constants (*K*_a_s) are: 70–240 M^−1^ (α-CD–2° amine TCA) [18,19]; (2.04–18.04) × 10^3^ M^−1^ (β-CD–2° amine TCA) [19,24]; 50–140 M^−1^ (α-CD–3° amine TCA) (Table S1); and (1.5–36.0) × 10^3^ M^−1^ (β-CD–3° amine TCA), Table S1. The distinct *K*_a_ values indicate that the α-CD complexes are at least two orders of magnitude less stable than the β-CD complexes, and the more hydrophobic 3° amine TCAs form a more stable complex with β-CD than do the 2° amine TCAs. By contrast, α-CD complexes with 2° and 3° amine TCAs are comparatively stable. Exceptions are β-CDs complex with PRT [21], DPM [26,27], and CPM [29], of which the aromatic A/B rings are nearly solely embedded in the CD cavity with the UV-derived *K*_a_s of (14.2–17.3) × 10^3^, 8.92 × 10^3,^ and (9.42–9.58) × 10^3^ M^−1^, respectively (Table 1 and Table S1). Note that *K*_a_s of the same complex with the same inclusion mode deduced from various techniques are rather different, particularly for the β-CD–3° amine TCA complexes (Table S1). In addition, the *K*_a_ values of β-CD–DPM [24,25], β-CD–NRT [22], β-CD–IPM [25], and β-CD–DXP [30] are more or less underestimated when compared to other studies.

The CD encapsulation of PRT and MPL has received lesser attention when compared to other TCAs. β-CDs (native and carboxymethyl and hydroxypropyl derivatives) have been efficiently applied as buffer additives in the capillary electrophoretic separation of TCAs, including PRT and MPL [31,32]. β-CD, dimethyl-β-CD (DIMEB) and PRT freebase form a rather stable equimolar inclusion complex, as inferred from the UV, circular dichroism, fluorescence, and ^13^C-NMR spectral changes upon the inclusion of the aromatic A/B-ring (totally) and the -CH=CH- group (partly) in the CD cavity [21]. The *K*_a_ values estimated from UV data are 14.2 × 10^3^ and 17.3 × 10^3^ M^−1^ for the β-CD–PRT base and DIMEB–PRT base complexes, respectively [21]. However, the solid-state β-CD–PRT inclusion complex could not be prepared for further analysis [21].

Both PRT and MPL are less flexible due to the -CH=CH- group bridging both wings and the -CH_2_–CH_2_- flexure across the central 6-membered C-ring, respectively (Scheme 1). Overall comparison among the 2° amine TCA complexes (Table 1), the β-CD–PRT complex is the most stable with *K*_a_s of (14.2–24.0) × 10^3^ M^−1^ [19,20,21], whereas the β-CD–MPL complex is the least stable with *K*_a_s of (4.8–4.9) × 10^3^ M^−1^ [19,20]. This suggests that the β-CD complexation stabilities are strengthened by the presence of PRT -CH=CH- bridge and weaken by the existence of MPL -CH_2_–CH_2_- flexure. The enhanced molecular stability of TCAs via CD encapsulation helped to reduce the side effects of drugs. For example, the hemolytic erythrocyte induced by TCAs is alleviated via CD encapsulation of IMP [33] and PRT [21]. The more stable the CD–IMP complexes, the greater the reduction in side effects, in accordance with the UV-derived *K*_a_s for β-CD, 2100 M^−1^ > γ-CD, 830 M^−1^ > α-CD, 50 M^−1^ [33]. Therefore, it is of interest to rationalize the inclusion complexation of β-CD–PRT and β-CD–MPL by in-depth structural investigation.

(ii) We have initiated a research project on the X-ray crystallographic analysis of the β-CD–TCA inclusion complexes for the past five years, around the 4th quarter of 2016. At that time, no crystal structure of CD inclusion complex with TCA was reported. We therefore decided to address the research gap as reviewed above. Thus far, there have been six β-CD–TCA inclusion complexes characterized crystallographically [23,28,34,35]; see the summary in Table 1 and Table S1. TCAs DPM, IPM, NRT, and AMT with the reflection symmetric wings prefer the inclusion mode of the aromatic A-ring in the β-CD cavity [23,28] while TCAs CPM and DXP with the reflection asymmetric wings/body of the (6-7-6)-tricyclic core favor the inclusion modes of the aromatic B-ring (without Cl) and the disordered A/B-ring in the β-CD cavity, respectively [35], Scheme 1 and Table 1. The topological inclusion structures of the six complexes crystallized in the same symmetry of the monoclinic system, space group *P*2_1_ have been thoroughly discussed [35]. However, after re-reviewing the literature, we found that the central C-ring could be altered for the significant changes of host–guest binding affinities of both the β-CD–PRT and β-CD–MPL complexes as described above.

Altogether, several questions remain about the inclusion complexation of β-CD–PRT (**1**) and β-CD–MPL (**2**), the published data remain scarce and disputable, especially the atomic-level characteristics derived crystallographically. Here, we hypothesized that (i) in the solid state, the structural changes owing to the -CH=CH- bridge in PRT and the -CH_2_–CH_2_- flexure in MPL could alter the inclusion complexation with β-CD structurally and energetically, as spectroscopically evidenced in solution. (ii) The structures and energies of both complexes are maintained through the proper inclusion geometry and host–guest interactions. To validate the two-fold hypothesis above, we explore to what extent the molecular and crystal structures of **1** and **2** are affected by the rigidity of the (6-7-6)-tricyclic core of PRT and the (6-6-6-6)-tetracyclic core of MPL via a systematic investigation using X-ray crystallography and DFT calculation.

## 2. Results and Discussion

**Figure 1:**
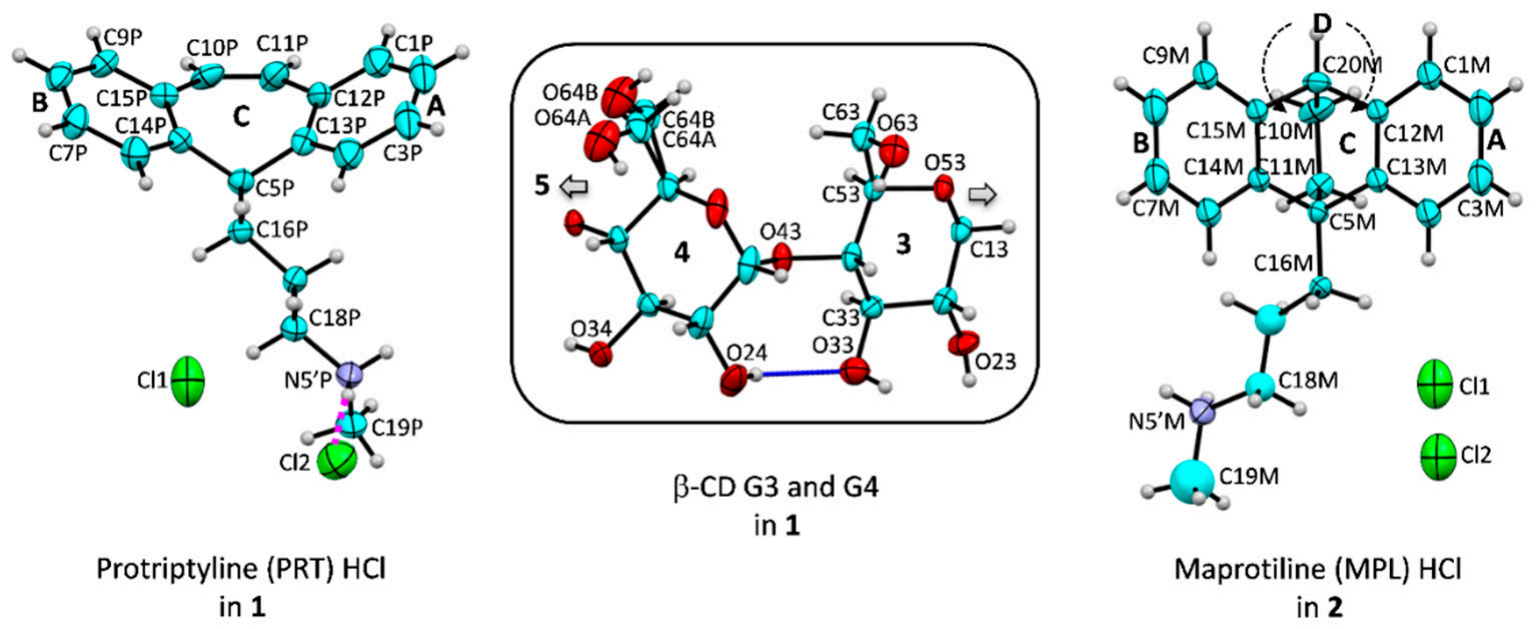
Atom numbering schemes of PRT HCl, MPL HCl, and β-CD (ORTEP plots at 20% probability level). In **1** and **2**, the protonated PRT-H^+^ and MPL-H^+^ at N5’ are charge-balanced by two half-occupied chlorides, of which their positions are arbitrarily shown here, except for PRT-H^+^ that is directly coordinated by chloride Cl2 (magenta dotted line). The intramolecular, interglucose O3(*n*)···O2(*n* + 1) hydrogen bonds stabilize the round β-CD conformation (connecting blue line). Note that the upward-facing obtuse butterfly angle has the (6-7/6-6)-tricyclic core lining with the B-C-A-rings from left to right, the -CH_2_–CH_2_- flexure, and the D-rings are beneath the C-ring for MPL.

β-CD nomenclature is used conventionally for carbohydrates, i.e., atoms C64A(B)–O64A(B) denote the methylene C6–H_2_ linked with the hydroxyl O6–H groups that are doubly disordered in sites A and B of glucose unit 4 (G4) in the β-CD–PRT HCl complex (**1**). As in our previous works on TCAs [23,28,35], atom numberings of PRT and MPL are used accordingly and further arbitrarily labeled with letters P and M, respectively (Figure 1). We organize our comprehensive discussion as follows: the inclusion complexation of β-CD–PRT (**1**) and β-CD–MPL (**2**) driven by induced-fit is described in detail for host β-CD and guests PTR, MPL in respective Section 2.1 and Section 2.2. Besides the distinct molecular structures of **1** and **2**, the different 3D arrangements are compared in Section 2.3. In final Section 2.4, the β-CD–TCA inclusion complexation investigated in the research project is thermodynamically rationalized by DFT-derived complexation energies and HPLC-, UV-derived binding constants.

### 2.1. PRT and MPL Are Conformationally Distinct in the β-CD Cavity Confinement

In attempts to search for TCA drugs whose side effects have been relieved by the β-CD encapsulation and their structural components are different from most TCAs, we come across the inclusion complexes of PRT and MPL, which are on the opposite side of the β-CD–TCA stability spectrum. The former is most stable [20,21], while the latter is least stable [20], based on the binding constants derived in solution. We envisage that the complex stability distinction is due to the -CH=CH- bridge in PRT and the -CH_2_–CH_2_- flexure in MPL, which are the most different structure portions compared to other TCAs; see Scheme 1 and Figure 1.

It is well known that in the less dense state (solution), the TCA side-chain is included in the β-CD cavity more thermodynamically stable than the aromatic ring; see the introduction. In the dense state of solids where the intermolecular interactions exist, the inclusion mode of an aromatic ring is exclusively observed as evidenced crystallographically [23,28,35]. Principally, the reflection symmetric A and B wings of the bending tricyclic core are equally entrapped in the β-CD cavity. However, scrutinizing the six reported β-CD–TCA crystal structures [23,28,35] revealed that the A-ring is more predominantly enclosed in the β-CD cavity over the B-ring. Consequently, the side-chain is folded to the opposite side (B-ring) to allow more open space in between for less steric hindrance and facilitate the total inclusion of the A-ring in the β-CD cavity [23,28,35]. In all, the central C-ring is placed nearby the O2–H/O3–H edge and partially embedded in the β-CD cavity. A question arises whether the inclusion mode is preserved if the C-ring at the bending point of the TCA molecule is structurally modified. The question has been fully addressed below.

**Figure 2:**
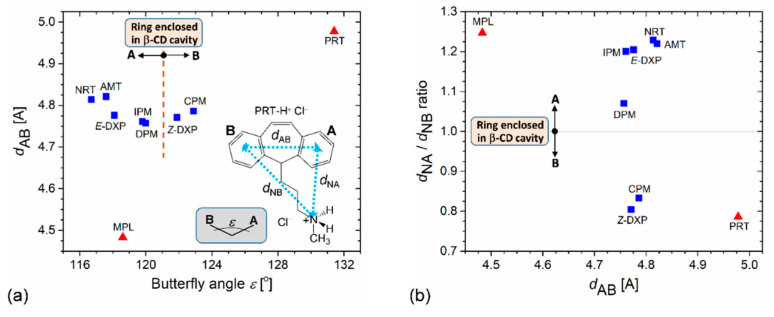
Scatter plots of (**a**) butterfly angle *ε* vs. distance *d*_AB_ and (**b**) distance *d*_AB_ vs. *d*_NA_/*d*_NB_ ratio of PRT (**1**) and MPL (**2**) in comparison to six β-CD–TCA complexes (NRT, AMT, DPM, IPM, CPM, and *E*/*Z*-DXP) [23,28,35] are shown in red triangles and blue squares. All TCA drugs are in HCl salt form. For more details, see Table 2. The TCA A/B-ring enclosed in the β-CD cavity is emphasized. The inset in (**a**) gives the chemical structure of PRT-H^+^ Cl^−^ and the definitions of relevant distances and the butterfly angle. The estimated uncertainties of angle and distance are about the legend width and one-half legend width, respectively.

Expectedly, in **2**, the -CH_2_–CH_2_- flexure incorporated across the central 6-membered C-ring caused a drastic change of flexibility at the central C-ring due to the bicyclo[2.2.2] octadiene skeleton. The central C-ring [C5M–C13M–C12M–C20M–C15M–C14M] and two fused D-rings [C5M–C11M–C10M–C20M–C12M(C15M)–C13M(C14M)] adopt a normal boat conformation with comparable puckering parameters: *Q*, 0.786(7)–0.834(8) Å; *θ*, 87.1(6)°–89.9(5)°; and *φ*, 2.3(5)°, 179.9(5)°, 358.4(4)° [36]. This narrows to the bending of the molecule, giving rise to the coincidence of the smallest butterfly angle (*ε*) and shortest centroid-centroid distance of the A and B rings (*d*_AB_); see the scatter plot in Figure 2a. By contrast, in **1**, the -CH=CH- bridge of the saddle-shaped cycloheptatriene C-ring enlengthens the *d*_AB_ distance and widens the *ε* angle of PRT simultaneously, thus maximizing both parameters (Figure 2a). The corresponding values of *ε* and *d*_AB_ are 131.4(4)°, 4.978 Å (**1**); 118.6(3)°, 4.473 Å (**2**); and 116.7(3)°–122.9(5)°, 4.757–4.821 Å for six complexed TCA [23,28,35], Table 2 and Figure 2a. Plus, other parameters describing the tricyclic core include annellation angle (*η*), twist angle (*τ*), and torsion angle C15–C10–C11–C12 (Table 2). The PRT -CH_2_=CH_2_- bridge (**1**) makes the tricyclic core less flexible as indicated by torsion angles C15–C10/O11–C11–C12 of about null, 1.4(19)° for **1** and –56.9(23)° to 69.9(14)° for other complexed TCAs [23,28,35]. The MPL -CH_2_–CH_2_- flexure (**2**) makes the tricyclic core most rigid among the complexed TCAs. This is evidenced from two relevant angles *η*, *τ*: 2.5(3)°, 1.7(3)° for **2**; 26.1(1), –178.3(4) for **1**; and 18.7(5)–28.9(3)°, –11.5(8)° to 10.3(5)° for other complexed TCAs [23,28,35].

The conformational distinction of TCAs is more pronounced for their flexible alkylammonium side chains (Table 2 and Figure 2b). Because the aromatic A-ring is favorably included in the β-CD cavity, the side-chain is folded to the same side of the B-ring. This is reflected by the ratio of the distances of N5’ to the A- and B-ring centroids (*d*_NA_/*d*_NB_) greater than 1 for most TCA complexes, including MPL (**2**), Table 2 and Figure 2b. Exceptions are PRT (**1**), CPM, and *Z*-DXP [35], of which the B-ring is embedded in the β-CD cavity, and the *d*_NA_/*d*_NB_ ratios are less than 1. Moreover, the structural flexibility of MPL is echoed by comparing with relevant crystal structures deposited in both major databases for small molecules [37] and macromolecules [38]. Surprisingly, no crystal structure of complexed or uncomplexed PRT (HCl or freebase) has been reported thus far. Only two crystal forms (A and B) of PRT HCl characterized by distinct powder X-ray diffraction patterns have been patented [39]. MPL is only found to exist in ammonium carbamate salt hemihydrate [40,41]. This structural survey implied that both PRT and MPL are rather labile and could be stabilized via inclusion complexation; see below.

**Figure 3:**
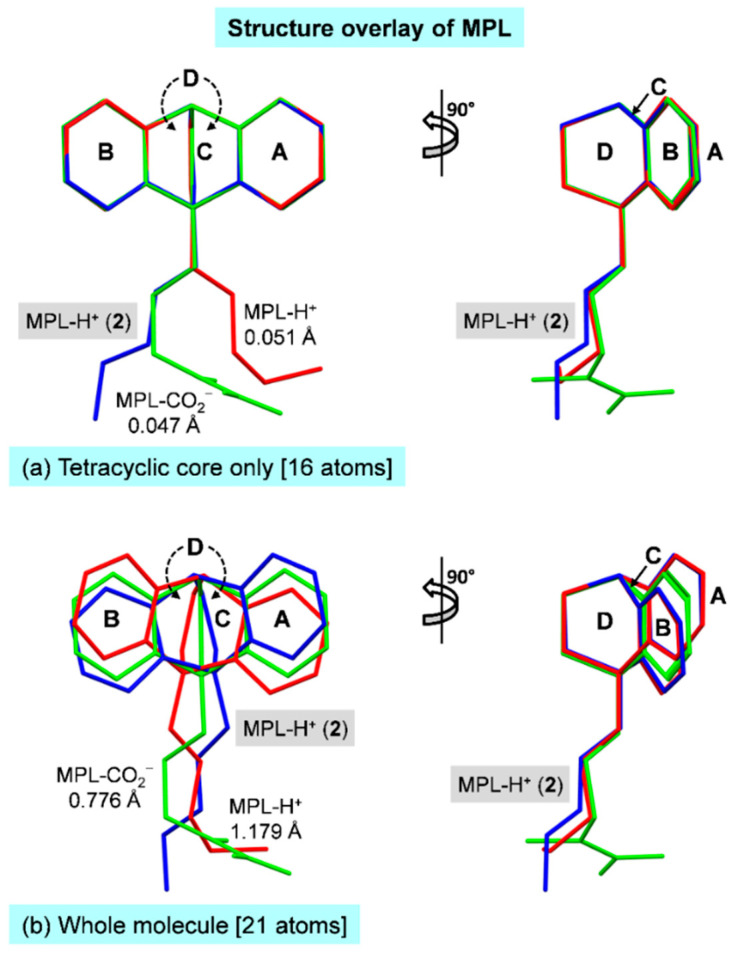
Rigid tricyclic core and flexible side-chain of TCAs are reflected by the structure overlays of protonated MPL in complex with β-CD (**2**) and maprotilinium carbamate hemihydrate (MPL-H^+^ and MPL-CO_2_^−^) [41], (**a**) the tetracyclic core and (**b**) whole molecule of MPL are included for the rms fit calculations.

Because the two crystal structures of maprotilinium carbamate hemihydrate reported in 1995 and 2019 are very similar [40,41], the latter is more accurately determined and hence used for the comparison. Figure 3a,b depicts the rigidity of the tetracyclic core and the flexibility of the side-chain of the protonated MPL in complex with β-CD (**2**) and with carbamate salt [41]. Clearly, the whole MPL-H^+^ molecules cannot be overlayed while the tetracyclic cores are nearly isostructural and perfect superimposable, as indicated by the rms fits of 0.776–1.179 Å and 0.047–0.051 Å, respectively. TCAs are conformationally flexible for their pharmacological activities, as evidenced from our insightful structural comparisons of TCAs in distinct lattice environments covering the uncomplexed TCA HCl salt, TCAs encapsulated in the carrier CD cavity, and TCAs in action while complexed with neurotransmitter transporter proteins [35].

**Figure 4:**
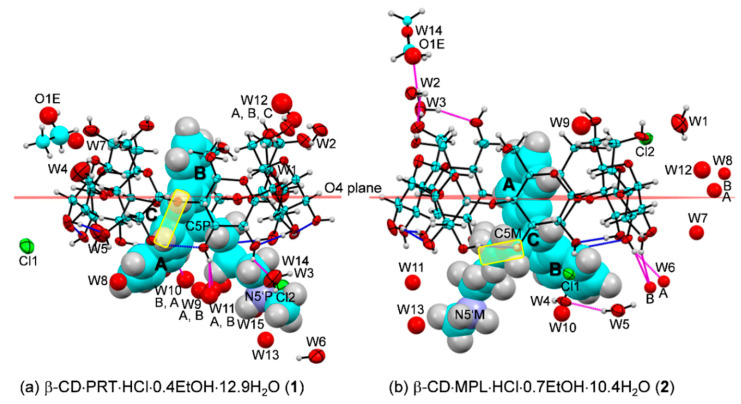
β-CD inclusion complexes with (**a**) PRT **1** and (**b**) MPL **2** in the solid state at 296 K (ORTEP plots at 20% probability level). For clarity, the drug molecules are shown in the space-filling model. The O–H···O hydrogen bonds stabilizing the round β-CD and the asymmetric unit are indicated by blue and magenta connecting lines, respectively. The -CH=CH- bridge of PRT and the -CH_2_–CH_2_- flexure of MPL at the central C-ring are emphasized in yellow areas. Note the reverse inclusion modes of PRT B-ring and MPL A-ring in the β-CD cavity.

The greater differences of PRT and MPL molecular bending (*e*) from other TCAs result in distinct inclusion structures and stabilities. The wider butterfly angles of TCAs, the deeper insertion of the aromatic ring in the β-CD cavity. This is indicated by larger centroid-centroid distances of A and B rings to β-CD O4-centroid of ~0.8–1.0 Å and the corresponding interplanar angles of 88°–90°, Table 2 [23,28,35]. The PRT B-ring (**1**) inserts deepest in the β-CD cavity (1.384 Å above the O4 plane) and makes a perfect right against the β-CD O4 plane, 89.7(2)°. By contrast, the MPL A-ring (**2**), DPM B-ring [28] are inclined by 85.4(2)°, 83.7(2)° with respect to the O4 plane and are placed 0.549, 0.467 Å above the O4 plane (Figure 4 and Table 2). Both **1** and **2** are stabilized by intermolecular interactions of types C–H···π (host–guest contacts in the asymmetric unit) and N5’–H···O2/O3/O6, O6–H···N5’ (crystal contacts are considered), Table 3, as observed in our earlier works [23,28,35]. The thermodynamic stabilities of **1** and **2** in comparison to the other six TCA complexes are discussed in Section 2.4.

### 2.2. Structural Adaptability of β-CD Macrocycles to the Inclusion of PRT and MPL

The β-CD inclusion complexes receive most research attention, among various CDs; see the example of CD–TCA complexes in Table 1 and Table S1. This is due to the optimal ring size and amphiphilic property of host β-CD for anchoring different guest molecules containing the aromatic moiety as evidenced crystallographically [37]. β-CD is not only an inexpensive, powerful encapsulating agent but also a biocompatible material [13]. In our research project on a comprehensive structural investigation of the β-CD–TCA inclusion complexes, although the TCA chemical structures are highly similar, their associations with β-CD differ in detail for both host and guest molecules from one complex to another. In particular, the most stable β-CD–PRT [21] and the least stable β-CD–MPL [20], it is noteworthy to what extent host β-CD and guest PRT, MPL molecules are changed structurally upon inclusion complexation to comply the induced-fit principle [42]. This has been addressed below and in Section 2.1 above.

**Figure 5:**
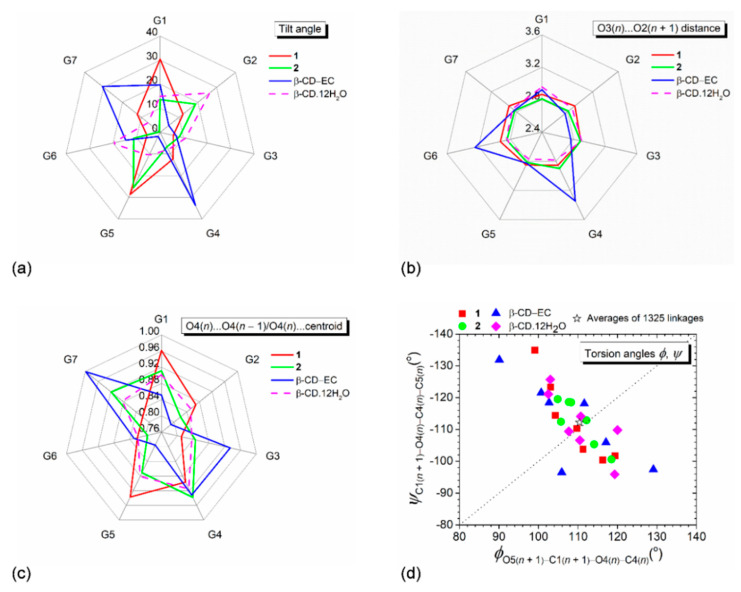
Radar plots of (**a**) tilt angles, (**b**) O3(*n*)∙∙∙O2(*n* + 1) distances, and (**c**) O4(*n*)∙∙∙O4(*n* − 1)/O4(*n*)∙∙∙centroid distance ratios of the β-CD seven glucose units (G1–G7) affected by the inclusion of the aromatic moieties of the PRT B-ring (**1**) and the MPL A-ring (**2**). (**d**) Scatter plot of torsion angles *φ*, *ψ* around glycosidic O4. Data of the inclusion complex β-CD–(–)-epicatechin (EC) [44] and the uncomplexed β-CD·12H_2_O [45] are given for comparison. Angles and distances are in ° and Å.

Generally, the conical annular shape of CDs comprises two main structure portions, the quite rigid glucose ring skeleton and the flexible O6–H groups, which are described by several parameters, as listed in Table S2, and illustrated in Figure 5. The CD structural parameters can be categorized into two groups. (i) The round CD conformation is primarily explained by the glucose tilt angle, the interglucose O3(*n*)···O2(*n* + 1) distance, and the parameters related to glycosidic O4, including the distance ratio of O4(*n*)···O4(*n* − 1)/O4(*n*)···centroid and the endocyclic torsion angles *φ* [O5(*n* + 1)–C1(*n* + 1)–O4(*n*)–C4(*n*)], *ψ* [C1(*n* + 1)–O4(*n*)–C4(*n*)–C5(*n*)] around glycosidic linkages [43]. (ii) The orientation of O6–H groups is described by the exocyclic torsion angles *χ* [C4–C5–C6–O6] and *ω* [O5–C5–C6–O6].

The orientation of O6–H groups with respect to the CD central cavity is influenced by the entrapped guest, the symmetry-related OH groups, and the surrounding solvent molecules. For the round β-CD·12H_2_O [45], three diametrically opposed glucose units are more inclined, and their O6–H groups point toward the cavity to hydrogen bond with water molecules (Table S3). Upon complexation, the TCA aromatic ring enters from the O2–H/O3–H side and occupies the β-CD cavity, and water molecules and O6–H groups are mostly repelled out of the cavity. Consequently, 12 out of 15 O6–H groups (7/8 for **1** including the two-fold disordered O64–H and 5/7 for **2**) point outward the cavity with torsion angles, *χ*, 47.7–77.7° and *ω*, −56.6° to −71.4° (Table S3). However, 3 out of 15 O6–H groups (O67 (**1**) and O62, O66 (**2**)) still direct inward the β-CD cavity, as indicated by the almost straight torsion angles *χ*(~±180°) and the acute torsion angles ω (60°–70°), Table S3. These O6–H groups actively participate in the H-bond networks stabilizing the crystal lattices of both complexes. Given examples are O67–H···O35–H···O26–H···Cl1···H–O22···H–O31···H1–N5’P (**1**, Table S4); N5’M–H1···O62–H···O4W–H2···O37; O66–H···O32–H···O23–H···Cl1 (**2**, Table S5). Note that β-CD O6–H groups do not make H-bonding with the embedded TCA aromatic ring, thus not stabilizing the β-CD–TCA inclusion complexes. On the contrary, the β-CD–polyphenol complexes are stabilized through O–H···O H-bonds between the enclosed catechol moiety and β-CD O6–H and water molecules [46]. The stabilization energies between two groups of complexes are compared in Section 2.4.

From free host to **1** and **2**, β-CDs are slightly distorted round due to the rigidity of seven glucose units. The ^4^*C*_1_ chair conformations of seven D-glucose residues interconnected via α-1,4-glycosidic bonds are mostly intact by total or partial inclusion of various guests with different sizes and shapes in the β-CD cavity, as indicated by the small oscillations of glucose puckering parameters *Q*, *θ* [36], Table S3. The corresponding values are: 0.552–0.571 Å, 1.4°–6.9° for **1**; 0.537–0.569 Å, 0.0°–7.9° for **2**; 0.559–0.583 Å, 1.4°–7.6° for uncomplexed β-CD [45]; 0.543–0.592 Å, 1.8°–9.7° for protocatechuic aldehyde (total inclusion, round β-CD) [46]; and 0.545–0.582 Å, 3.2°–8.8° for (–)-epicatechin (EC) (partial inclusion, highly distorted β-CD) [44]. β-CD hydrate is more rigid compared to other family members. Due to the optimal CD ring strain for thermodynamic stability, α-CD with six glucose units existing in a ^4^*C*_1_ chair form is the stable smallest natural occurring CD. However, recently, the three-glucose-composing CD has been synthesized and characterized crystallographically, revealing that one glucose is conformationally changed to ^5^*S*_1_ screw form to reduce ring strain [47].

At this point, what are the parameters better differentiating the roundness degree of CD structures? The answer to this question is displayed in Figure 5a–d. The two diametrically opposed glucose units of β-CDs (G1, G5 in **1** and G2, G5 in **2**) are more inclined (28.7°–30.4° and 18.8°–25.5°) to grip the PRT B-ring and MPL A-ring, giving rises to distorted round β-CD in **1** more than **2** (Figure 5a, Table S3). β-CDs in **1** and **2** remain more or less round because the O3(*n*)···O2(*n* + 1) belt (i.e., the systematic interglucose O3(*n*)···O2(*n* + 1) H-bonds) securing the β-CD round conformation is not disrupted from the large glucose inclination, 26°–30°. The O3(*n*)···O2(*n* + 1) distances of the three β-CDs fall in a short range of 2.770–2.926 Å (Figure 5b and Table S3). By contrast, the tighter fit of tea EC resorcinol moiety in the β-CD cavity requires larger tilt angles (30.6°–33.7°) and longer O3(*n*)···O2(*n* + 1) distances (3.246–3.346 Å) of two diametrically opposed glucoses [44]. This breaks the belt of O3(*n*)···O2(*n* + 1) H-bonds, resulting in the large distortion from a round conformation [44]. Plus, the CD roundness is judged from the O4 relevant parameters, i.e., (i) the ratio of O4(*n*)···O4(*n* − 1) to O4(*n*)···centroid distances, and (ii) the perpendicular diagonal distribution (PDD) and the sum of averages of torsion angles *φ*, *ψ*. Clearly, the radar plots of the adjacent O4/O4···centroid distance ratios (0.868 for a perfect heptagon) have similar spike (peak) positions and show the degree of roundness as follows: uncomplexed β-CD [45] > **2** > **1** > β-CD–EC complex [44], Figure 5c. This agrees with the extent of PDD of *φ*, *ψ*, that is, the shorter PDD, the rounder β-CD structure (Figure 5d). Moreover, the number of data points distributed above and below the diagonal line are comparable, yielding the sum of averages of *φ*, *ψ* about null. Statistical analysis revealed that the averages of *φ*, *ψ* from 1325 linkages of β-CDs give a sum of practically zero (marked with a star nearly on the diagonal line in Figure 5d), indicating the CD close ring structure [43].

**Figure 6:**
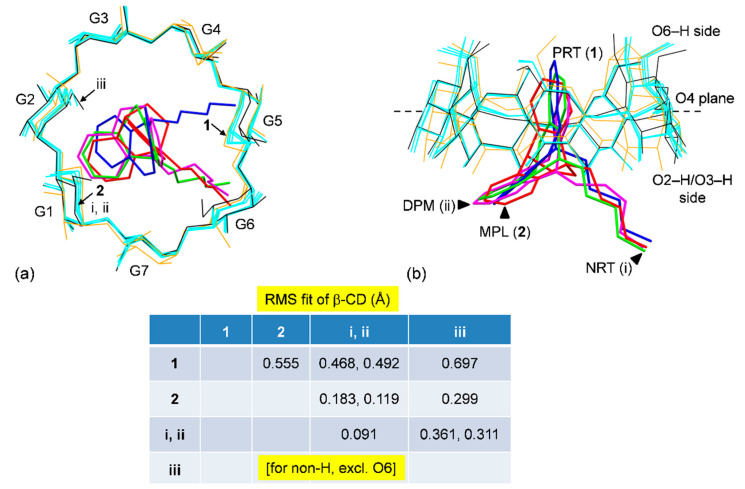
Structure superpositions of four β-CDs (orange and cyan wireframes) in complex with various 2° amine TCAs (sticks), PRT (**1**; blue), MPL (**2**; red), NRT (i; green), DPM (ii; magenta) [23,28], and in the uncomplexed β-CD·12H_2_O (iii; black) [45], viewed from (**a**) the top and (**b**) the side. The rms fits are given in the middle inset.

Overall, upon the inclusion of 2° amine TCAs including PRT B-ring (**1**), MPL A-ring (**2**), NRT A-ring (i; [23]), DPM A-ring (ii; [28]), β-CDs are different as indicated by the rms fits in the range of 0.468–0.555 Å; **1** is a reference structure (Figure 6). A larger difference is obtained when compared β-CD in **1** to β-CD·12H_2_O (iii; [45]); rms fits of 0.697 Å. When β-CD (**2**) is compared to β-CDs (i–iii), the rms fits are in a narrow span of 0.119–0.299 Å, suggesting β-CD (**2**) is rounder than β-CD (**1**), Figure 6. Note that the rms fit computed for each structure pair considers all structure components concurrently, excluding the rotatable O6–H groups and H atoms, which is more understandable than comparing each structure moiety separately, as discussed above. Plus, β-CD adapting for inclusion complexation with PRT is obviously observed. In Section 2.4, we explain further that to what extent the complex stability of β-CD–PRT is compared to those of other β-CD–TCA complexes.

### 2.3. PRT Ethylene Group (1) Makes the Distinction in the Complex 3D Arrangements

**Figure 7:**
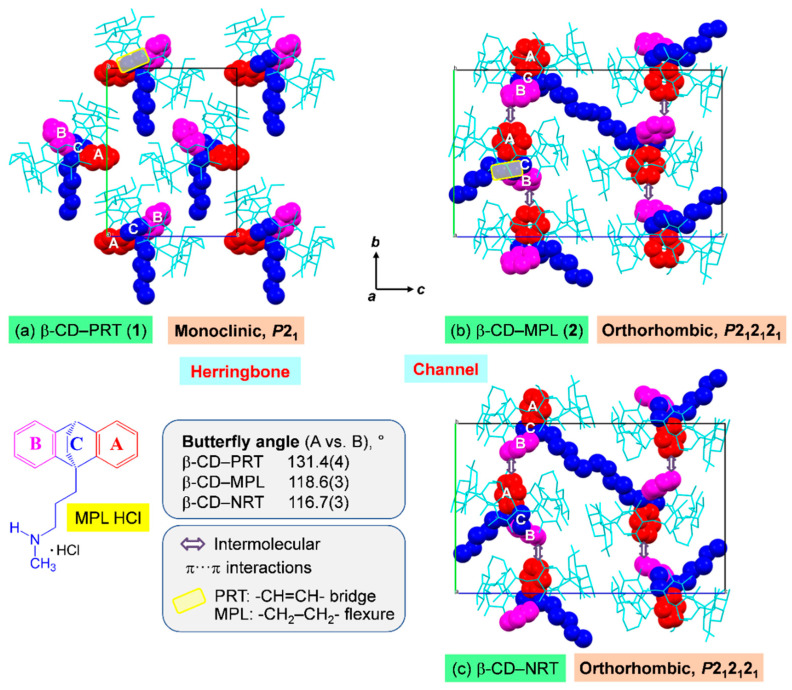
3D arrangements of the inclusion complexes (**a**) β-CD–PRT HCl in monoclinic, *P*2_1_ (**1**) existing in the herringbone pattern, (**b**) β-CD–MPL HCl (**2**) and (**c**) β-CD–NRT HCl [23] both packing in the channel-type structure stabilized by intermolecular edge-to-face π∙∙∙π interactions. β-CD, PRT, MPL, and NRT molecules are shown with cyan wireframes and space-filling models (A-ring = red; B-ring = magenta; C-ring and side-chain = blue). Water molecules, chlorides, and H atoms are omitted for clarity. Examples of the PRT -CH=CH- bridge and the MPL -CH_2_–CH_2_- flexure at the central C-ring are emphasized in yellow areas. Note the reverse inclusion modes of PRT B-ring and MPL A-ring in the β-CD cavity. TCAs composing the B-C-A rings and the side-chain are shown as an example for MPL HCl, and the butterfly angles are compared in the inset.

In solution, the β-CD encapsulation of the TCA side-chain is predominantly observed (Table 1), and the O6–H∙∙∙N5′ H bond and O2/O3–H∙∙∙π interactions are vital intermolecular forces, as demonstrated in our previous work on the DFT calculation of the β-CD–DPM/IPM complexes in the gas phase (disregarded the solvent effect) [28]. However, upon crystallization via evaporation of the solvent (aqueous EtOH), various host and guest molecules are in intermolecular contacts, forming complex nuclei, packing tightly and growing into stable single crystals of the β-CD–TCA complexes with the A-ring (mostly selectively) or the B-ring (occasionally) entrapped in the β-CD cavity [23,28,35]. The TCA aromatic A/B-ring is primarily maintained in position by host–guest C–H∙∙∙π interactions. Moreover, the crystal contacts of types N5’–H∙∙∙O H-bonds (host–guest), O–H∙∙∙O H-bonds (host–host and via bridging water), and edge-to-face π∙∙∙π interactions (guest–guest) help to stabilize the crystal lattice. This observation is true for the previous six β-CD–TCA complexes and the present complex of MPL (**2**), Tables S4 and S5. These complexes belong to the same crystal symmetry of the orthorhombic system, space group *P*2_1_2_1_2_1_ and have comparable unit cell parameters. They are packed in a channel-type structure as shown for **2** and the β-CD–NRT complex [23], where both drugs have comparable butterfly angles (Figure 7b,c). Note that although the -CH_2_–CH_2_- flexure affects the inclusion structure and stability of β-CD–MPL (see Section 2.1 and Section 2.4), it does not influence the overall packing of a channel-type structure (Figure 7b,c).

On the contrary, **1** crystallizes in the monoclinic space group *P*2_1_ and thus has a unique packing scenario. The -CH=CH- bridge restricts the vertical motion of the central C-ring, thus widening the butterfly angle and simultaneously enlengthening the centroid-centroid distance of A and B rings to the maximum values of 131.4(4)° and 4.978 Å, respectively (Table 2). As a result, the PRT B-ring is perfect vertically aligned and is deepest embedded in the β-CD cavity (Section 2.2). PRT molecules are individually included in the symmetry-related β-CD cavities and are not in intermolecular contact with other PRT molecules (Figure 7a). This facilitates the distinct 3D arrangement of a herringbone pattern, a typical packing structure of CD inclusion complexes with small guest molecules isolated enclosed in the CD cavity [48]. Note that the main difference of crystal contacts attributed to the distinct packing structures of **1** and **2** is the guest–guest edge-to-face π∙∙∙π interactions, which are absent from **1** (Figure 7a,b and Tables S4 and S5).

### 2.4. Different Inclusion Stability of 1 and 2 in Solution and Gas Phase

Supramolecular CD inclusion complexes are an excellent paradigm of the induced-fit effect [42]. Both host and guest molecules affect each other and adapt themselves to an extent for space-complimentary and optimal intermolecular interactions (e.g., hydrogen bonds, van der Waals, etc.), establishing a thermodynamically stable inclusion complex. In Section 2.1 and Section 2.2, we fully discuss the structural changes of host β-CD and guests PRT, MPL owing to inclusion complexation. The amphiphilic β-CD and mostly hydrophobic TCAs are associated via intermolecular interactions, C–H∙∙∙π (primarily within the host–guest asymmetric unit) and N–H∙∙∙O, O–H∙∙∙N (further forces from crystal contacts). Note that in the crystalline state where the inclusion mode of the aromatic A/B-ring is solely observed, the TCA alkylammonium side-chain residing outside the β-CD cavity, near the O2–H/O3–H rim does not interact with β-CD, it is therefore sensible to consider TCA in neutral form (not protonated state) for the evaluation of host–guest interactions and thermodynamic stabilization in the gas phase by DFT calculation [23,28,35].

**Figure 8:**
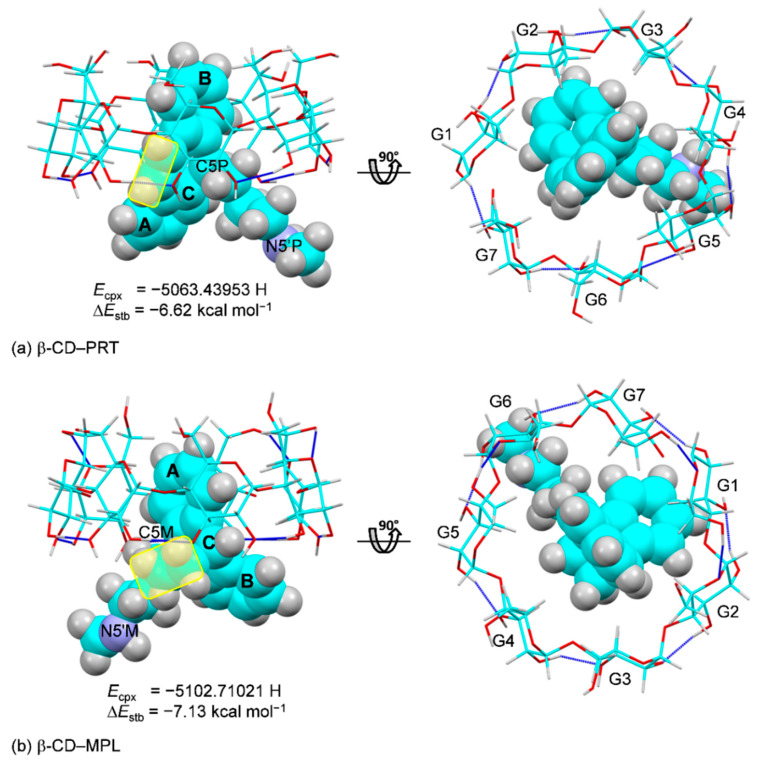
Inclusion complexes of (**a**) β-CD−PRT and (**b**) β-CD−MPL, derived from DFT complete-geometry optimization in the gas phase; side view (**left**) and top view (**right**). For comparison, the complex and stabilization energies (*E*_cpx_ and Δ*E*_stb_) are given; see also Table 3 and Tables S6 and S7. The belt of O3(*n*)∙∙∙O2(*n* + 1) hydrogen bonds stabilizing the round β-CD conformation are indicated by blue connecting lines. The PRT -CH=CH- bridge and the MPL -CH_2_–CH_2_- flexure at the central C-ring are emphasized in yellow areas.

The DFT-optimized structures in the vacuum of the β-CD–PRT and β-CD–MPL inclusion complexes are displayed in Figure 8a,b. They are similar to the X-ray-derived structures, as indicated by the respective rms fits of 0.333 and 0.344 Å; the illustrations are not shown here. This is because the belt of interglucose O3(*n*)∙∙∙O2(*n* + 1) H-bonds existing in the solid state is preserved in the gas phase. The vertically aligned PRT B-ring and MPL A-ring in the β-CD cavity are kept in place by weak intermolecular C–H∙∙∙π interactions with similar stabilization energies (Δ*E*_stb_s) of −6.62 and −7.13 kcal mol^−1^, respectively. This contrasts to what is observed in solution as the β-CD–PRT and β-CD–MPL complexes are on the opposite ends of the stability spectrum, i.e., the former (the most stable) and the latter (the least stable) [20]. The reason for this is the minimum and maximum interactions of MPL and PRT with the β-CD bonded stationary phase in the HPLC separation process of TCAs [20].

**Figure 9:**
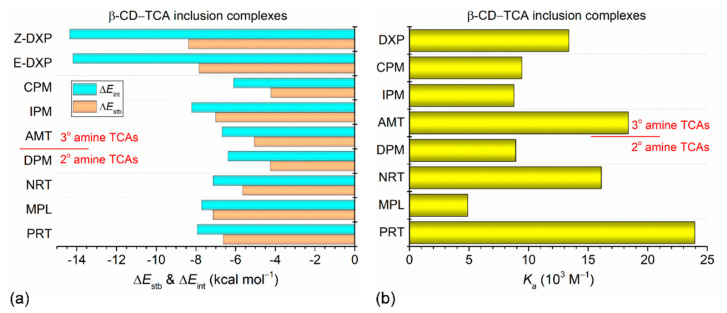
(**a**) Stabilization energy (Δ*E*_stb_) and interaction energy (Δ*E*_int_) of inclusion complexes β-CD–PRT base and β-CD–MPL base, in comparison to those of other six reported complexes β-CD–NRT base, β-CD–AMT base [23], β-CD–DPM base, β-CD–IPM base [28], and β-CD–CPM base, β-CD–*E*/*Z*-DXP base [35], derived from DFT full-geometry optimization in the gas phase; see also Table S7. (**b**) Stability constants (*K*_a_) are deduced from HPLC and UV data in solution; DXP denotes the racemic mixture of *Z*-DXP and *E*-DXP [20,27,29]. The estimated uncertainties of Δ*E* = 0.1 kcal mol^−1^ and *K*_a_ = 0.1×10^3^ M^−1^.

A question arises on how the stabilities of relevant β-CD–TCA complexes are theoretically compared. On the basis of the HPLC- and UV-derived *K*_a_ values (10^3^ M^−1^) in solution for β-CD complexes with the TCA aromatic A/B-ring enclosed in the cavity (Figure 9b, Table 1 and Table S1), the complex stability order is PRT, 24.0 > AMT, 18.4 > NRT, 16.1 > DXP, 13.4 > CPM, 9.4 > DPM, 8.9 ~ IPM, 8.8 > MPL, 4.9 [20,27,29]. The thermodynamic stability order based on Δ*E*_stb_s (in kcal mol^−1^) of the β-CD inclusion complexes in the vacuum is *Z*-DXP, –8.37 > *E*-DXP, –7.84 > MPL, −7.13 ~ IPM, –7.00 > PRT, –6.62 > NRT, –5.64 > AMT, –5.06 > DPM, –4.25 ~ CPM, –4.22 (Figure 9a and Table S7). The complex stability order based on interaction energies (Δ*E*_int_s) has a similar tendency (Table S7). The differences of stability orders (Δ*E*_stb_s vs. *K*_a_s) are probably due to the combination of the B3LYP functional with a small 6-31+G* basis set, and the mixed multi-inclusion modes likely exist in solution (*K*_a_s), but the X-ray-derived inclusion mode of the aromatic ring is solely considered in the gas phase (Δ*E*_stb_s and Δ*E*_int_s). Note that the Δ*E*_stb_s in the range of –4.25 to –8.37 kcal mol^−1^ indicate the weak intermolecular C–H∙∙∙π interactions of the β-CD–TCA complexes. On the contrary, the β-CD–polyphenol complexes with the catechol moiety embedded in the cavity and maintained in position through H-bonding with CD rims are ~2–4 times more stable; Δ*E*_stb_ = –14.38 to –32.58 kcal mol^–1^ [46]. This suggests that Δ*E*_stb_s and Δ*E*_int_s of the β-CD–TCA complexes are somewhat underestimated, and the approximations deserve further attention. Although the DFT calculations at the B3LYP/6-31+G*/4-31G level worked quite well for the H-bonded systems of β-CD–polyphenol inclusion complexes [46], the B3LYP/6-31+G*/4-31G method seemed to insufficiently describe the dispersion interactions predominantly present in the β-CD–TCA complexes.

**Figure 10:**
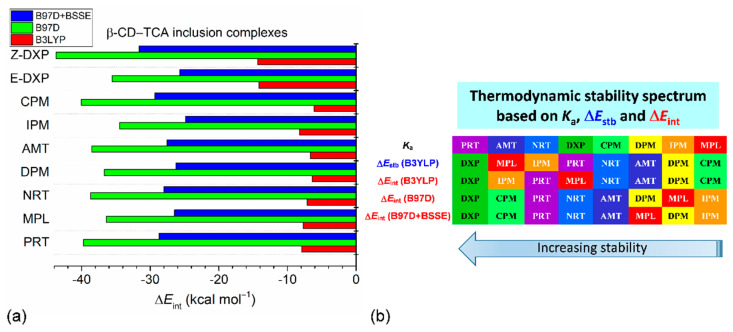
(**a**) Interaction energies (Δ*E*_int_s) of eight β-CD–TCA base inclusion complexes derived in the gas phase, with mixed basis sets 6-31+G*/4-31G using various DFT methods, that is, without dispersion correction (B3LYP), with dispersion correction (B97D), and with dispersion and BSSE corrections (B97D+BSSE). (**b**) Thermodynamic stability spectrum based on *K*_a_ [20,27,29], Δ*E*_stb,_ and Δ*E*_int_ of eight β-CD–TCA base inclusion complexes. DXP denotes the racemic mixture of *Z*- and *E*-enantiomers for *K*_a_ and the most stable complex of *Z*-DXP for Δ*E*. The estimated uncertainties of Δ*E* = 0.1 kcal mol^−1^; see also Figure 9 and Tables S7–S9.

Therefore, we further evaluated to what extent the dispersion forces affect the interaction energies of eight β-CD–TCA complexes. The structures optimized by the B3LYP/6-31+G*/4-31G method were employed to calculate single-point energies (Δ*E*_int_s) using the dispersion-corrected DFT with B97D/6-31+G*/4-31G approximation (Table S8). Clearly, after dispersion corrections, the magnitudes of Δ*E*_int_s(B97D) ranging from −34.48 to −43.72 kcal mol^−1^ increase ~2–6 times when compared to the uncorrected ones. The β-CD–TCA thermodynamic stabilities are (kcal mol^−1^) *Z*-DXP, –43.72 > CPM, –40.05 ~ PRT, –39.71 > NRT, –38.71 ~ AMT, –38.52 > DPM, –36.71 ~ MPL, –36.42 > *E*-DXP, –35.55 > IPM, –34.48 (Table S8), which are different from the stability orders mentioned above (Figure 10). When the BSSE correction was also taken into account (Δ*E*_BSSE_ = 9.44–12.08 kcal mol^−1^), the resulting Δ*E*_int_s(B97D+BSSE) decrease and fall in a smaller range, −24.88 to −31.63 kcal mol^−1^, but the stability order is mostly the same as the case of excluding the BSSE (Figure 10 and Table S9). Plus, larger basis sets 6-31G(d,p) and 6-311++G(2d,p) more or less change the values of Δ*E*_int_s(B97D) of the β-CD–*E*-DXP complex from −35.55 kcal mol^−1^ [6-31+G(d)/4-31G] to −35.26 and −28.65 kcal mol^−1^, respectively (Table S8).

## 3. Materials and Methods

### 3.1. Materials

β-CD (≥95%) was obtained from Cyclolab, Budapest, Hungary (code CY-2001). PRT HCl (≥99%; code P8813) and MPL HCl (≥98%; code M2527) were purchased from Sigma-Aldrich (Steinheim, Germany) and TCI Chemicals (Tokyo, Japan), respectively. Absolute EtOH (≥99.8%) was supplied by Liquor Distillery Organization, Excise Department, Thailand. All chemicals were used as received. The ultrapure water was provided by a Milli-Q Water System.

### 3.2. X-ray Crystallography

#### 3.2.1. Single-Crystal Preparation

As described in our previous work [35], slow solvent evaporation was employed for the crystallization of CD inclusion complexes. Homogeneous concentrated solutions of the 1:1 β-CD–PRT HCl (**1**) and β-CD–MPL HCl (**2**) inclusion complexes were prepared from the corresponding solid mixtures of β-CD 50 mg (0.044 mmol), PRT HCl 13.2 mg (0.044 mmol) and MPL HCl 13.8 mg (0.044 mmol) dissolved in 1 mL 50% (*v/v*) EtOH–H_2_O. Suitable quality single crystals grew after two weeks of solvent evaporation.

#### 3.2.2. X-ray Diffraction Experiment

Colorless rod-like single crystals of **1** and **2** were separately loaded into a thin-walled glass capillary (Hilgenberg, Germany). A number of crystals were screened for the consistency of unit cell parameters and sufficient diffracting power. Different crystals belonged to the monoclinic and orthorhombic systems and had comparable unit cell constants, suggesting two new inclusion complexes of **1** and **2**, respectively. Well-diffracting crystals of **1** and **2** were used for X-ray data collection at 296 K to 0.83-Å atomic resolution on a Bruker APEXII CCD area-detector diffractometer (MoKα radiation; *λ* = 0.71073 Å). Data processing assisted by the APEX2 software suite [49] was accomplished according to standard procedures, i.e., began with the integration of diffraction frames using SAINT [50], followed by scaling and multi-scan absorption correction using SADABS [49], and completed by merging with XPREP [50]. The total numbers of 42,205 and 53,243 independent reflections with nearly 100% coverage and *R*_int_ of 0.0354 and 0.0284 were obtained for **1** and **2**, respectively.

#### 3.2.3. Structure Solution and Refinement

The crystal structures of **1** and **2** were solved by the intrinsic phasing method with SHELXTL XT [49], providing all non-H atoms of β-CD, PRT, MPL, and most non-H atoms of solvent molecules. The two highest peaks were suitably assigned as two doubly disordered chlorides. The remaining non-H atoms with lower occupancy factors, including water and ethanol sites, were located by difference Fourier electron density maps graphically assisted by WinCoot [51]. The drug PRT and MPL in HCl salt forms retained in the solid state as protonated PRT-H^+^ and MPL-H^+^, which were net charge-balanced and indirectly linked by two-fold disordered chlorides (Cl1 and Cl2), as previously found in the crystals of TCA HCl in complex with β-CD [23,28,35]. Exception for **1** of which PRT-H^+^ was directly coordinated by a half-occupied chloride Cl2 in addition to the isolated Cl1, Figure 1.

Most non-H atoms were refined anisotropically by full-matrix least-squares on *F*^2^ using SHELXTL XLMP [49]. Exceptions for some C atoms of the side-chain of MPL (**2**) and partially occupied water and ethanol molecules, which were refined isotropically. H atoms of rigid groups were positioned geometrically and treated with a riding model: C−H = 0.93 Å, *U*_iso_ = 1.2*U*_eq_(C)(aromatic); C−H = 0.98 Å, *U*_iso_ = 1.2*U*_eq_(C)(methine); C−H = 0.97 Å, *U*_iso_ = 1.2*U*_eq_(C)(methylene); C−H = 0.96 Å, *U*_iso_ = 1.5*U*_eq_(C)(methyl); and N−H = 0.89 Å, *U*_iso_ = 1.2*U*_eq_(2° ammonium). The hydroxyl H atoms initially located by difference Fourier maps were refined using ‘AFIX 147’ or ‘AFIX 83’ with restraints O−H = 0.84 Å, *U*_iso_ = 1.5*U*_eq_(O). H atoms of highly occupied waters could be located by different Fourier maps. To prevent short H∙∙∙H distances in the refinement, BUMP antibumping restraints were applied. The refinement converged to final *R*_1_ = 0.0781 and 0.0768 for **1** and **2**, respectively. For more details of data collection and refinement statistics, see Table S1.

### 3.3. DFT Calculations

#### 3.3.1. Full-Geometry Optimization

Atomic resolution X-ray structures are accurately determined and are suitable initial structures, providing economical computing resources for DFT energy minimization, as demonstrated in our previous works on the β-CD inclusion complexes with TCAs [23,28,35]. Before the calculation, the underestimated X-ray-derived X–H bond lengths in the β-CD–PRT base and β-CD–MPL base inclusion complexes were normalized to neutron hydrogen distances: C–H, 1.083 Å; N–H, 1.009 Å; and O–H, 0.983 Å [52]. The corrected structures were optimized by the semiempirical PM3 method and then fully re-optimized by DFT calculation using the B3LYP functional in the gas phase with mixed basis sets 6-31+G* for H, N, O, and 4-31G for C. This was inspired by the DFT calculation on a large-ring CD with 26 glucose units [53]. Note that the full-geometry optimization by semiempirical PM3 method was frequently used prior to the DFT energy minimization because the PM3 method provided a suitable starting structure for the DFT calculation, especially when the X-ray-derived structures of relevant inclusion complexes were not available [54,55]. All calculations were carried out using program GAUSSIAN09 [56] on a DELL PowerEdge T430 server. Stabilization energy and interaction energy of the complex (Δ*E*_stb_ and Δ*E*_int_) were calculated using Equations (1) and (2).

Δ*E*_stb_ = *E*_cpx_ − (*E*_β-CD_opt_ + *E*_D_opt_) (1)

Δ*E*_int_ = *E*_cpx_ − (*E*_β-CD_sp_ + *E*_D_sp_) (2)

where *E*_cpx_, *E*_β-CD_opt,_ and *E*_D_opt_ are the molecular energies from full-geometry optimization of complex, host β-CD and drug PRT/MPL, respectively; *E_β_*_-CD_sp_ and *E*_D_sp_ are the corresponding single-point energies in the complexed states. The accuracy of the molecular self-consistent-field (SCF) energy = 0.1 kcal mol^−1^.

#### 3.3.2. Dispersion and BSSE Corrections

Moreover, to improve the DFT results, three approximations, including the basis set superposition error (BSSE) according to the counterpoise method [57], the dispersion-corrected functional B97D, and larger basis sets were further considered. The structures optimized from B3LYP/6-31+G*/4-31G were used to calculate single-point energies Δ*E*_int_s with the corrections of dispersion (B97D) and BSSE. Plus, to check the influence of basis sets on Δ*E*_int_s, we estimated Δ*E*_int_s of the β-CD–*E*-DXP complex from the B97D/6-31G(d,p) and B97D/6-311++G(2d,p).

## 4. Conclusions

Mental health concern over depression has risen tremendously due to the coronavirus disease 2019 (COVID-2019) pandemic, suggesting the requirement for efficient concurrent treatment of both depression and COVID-19. Tricyclic antidepressants (TCAs) are efficacious for the treatment of depression, albeit they have more side effects. Cyclodextrins (CDs) are powerful encapsulating agents for improving molecular stability, water solubility, and lessening undesired effects of drugs. Aiming at an in-depth atomic-level understanding of the β-CD–TCA inclusion complexation, we carried out a comprehensive study series via single-crystal X-ray diffraction and DFT full-geometry optimization. Here, we come to the β-CD encapsulation of protriptyline (PRT; **1**) with -CH=CH- group of the 7-membered C-ring bridging the aromatic A–B rings and maprotiline (MPL; **2**) with -CH_2_–CH_2_- flexure across the 6-membered C-ring. On the opposite ends of the β-CD–TCA stability spectrum are **1** and **2**, the most and least stable complexes, respectively, based on the binding constants (*K*_a_s) derived in solution.

X-ray crystallography unveiled that in the β-CD cavity, the PRT B-ring and MPL A-ring have a nearly perfect right angle alignment against the O4 plane. The PRT -CH=CH- bridge widens while the MPL -CH_2_–CH_2_- flexure narrows the butterfly angles. This facilitates the deepest and shallower insertion of PRT (**1**) and MPL (**2**) in the distorted round β-CD cavity for inclusion complexation, which is primarily stabilized by C–H···π interactions. This is indicated by DFT-derived complex stabilization energies (Δ*E*_stb_s), although the stability orders of the β-CD–TCA complexes based on *K*_a_s and Δ*E*_stb_s are different. The DFT results were improved by the dispersion and the basis set superposition error (BSSE) corrections. The distinctions between **1** and **2** are more pronounced in the crystal lattice. Without the PRT–PRT edge-to-face π···π interactions, **1** in the monoclinic, *P*2_1_ prefers a herringbone packing pattern, whereas the presence of MPL–MPL π··· π interactions, **2** in the orthorhombic, *P*2_1_2_1_2_1_ favors a channel-type packing structure as observed in all other complexes. This work provides the first crystallographic evidence of PRT and MPL stabilized in the β-CD cavity, facilitating the improvement of TCA bioavailability and usage and suggesting the potential application of CDs for efficient drug delivery. The COVID-19 pandemic and its mental effect (depression) bring us both concerns and opportunities. The combinatory treatment with antidepressants and COVID-19 medications could help to reduce the severity of COVID-19 [58,59,60].

## Figures and Tables

**Table 1:** Summary of the CD–2° amine TCA complexes characterized by various techniques.

Host	Guest	Ratio	Inclusion Mode ^a^		*K*_a_, M^−1^	Ref.
			Aromatic	Side-Chain	(Tech.) ^b^	
α-CD	PRT	1:1		✓	120 (FI)	[18]
α-CD	PRT	1:1		✓	0.11 × 10^3^ (I)	[19]
β-CD	PRT	1:1		✓	18.04 × 10^3^ (I)	[19]
β-CD	PRT	1:1	✓ A/B		24.0 × 10^3^ (H)	[20]
β-CD	PRT	1:1	✓ A/B		14.2 × 10^3^ (U)	[21]
β-CD	PRT	1:1	ND ^c^		ND (CD)	[21]
β-CD	PRT	1:1	✓ A/B		ND (F)	[21]
β-CD	PRT	1:1	✓ A/B		ND (N)	[21]
β-CD	PRT	1:1	✓ B		ND (X)	This work
β-CD	PRT	1:1	✓ B		ND (Tg)	This work
DIMEB	PRT	1:1	✓ A/B		17.3 × 10^3^ (U)	[21]
DIMEB	PRT	1:1	ND		ND (CD)	[21]
DIMEB	PRT	1:1	✓ A/B		ND (F)	[21]
DIMEB	PRT	1:1	✓ A/B		ND (N)	[21]
α-CD	MPL	1:1		✓	240 (FI)	[18]
α-CD	MPL	1:1		✓	0.12 × 10^3^ (I)	[19]
β-CD	MPL	1:1		✓	4.81 × 10^3^ (I)	[19]
β-CD	MPL	1:1	✓ A/B		4.9 × 10^3^ (H)	[20]
β-CD	MPL	1:1	✓ A		ND (X)	This work
β-CD	MPL	1:1	✓ A		ND (Tg)	This work
α-CD	NRT	1:1		✓	70 (FI)	[18]
α-CD	NRT	1:1		✓	0.09 × 10^3^ (I)	[19]
β-CD	NRT	1:1		✓	16.77 × 10^3^ (I)	[19]
β-CD	NRT	1:1	✓	✓	235 (U), 211 (F)	[22]
β-CD	NRT	1:1	✓ A/B		16.1×10^3^ (H)	[20]
β-CD	NRT	1:1	✓ A		ND (X)	[23]
β-CD	NRT	1:1		✓	ND (Tgl)	[23]
β-CD	DPM	1:1		✓	2.04 × 10^3^ (C)	[24]
β-CD	DPM	1:1	✓	✓	42.2 (U), 32.0 (F)	[25]
β-CD	DPM	2:1	✓ A + B		ND (F)	[26]
β-CD	DPM	1:1	✓		8.92 × 10^3^ (U)	[27]
β-CD	DPM	1:1	✓ A		ND (X)	[28]
β-CD	DPM	1:1	✓ A	✓	ND (Tg)	[28]

^a^ TCA moiety included in CD cavity: aromatic rings A, B, or side-chain. ^b^ Binding constant (*K*_a_) at 298 K derived from different techniques, mostly in solution: flow injection (FI); fluorescence (F); UV-vis (U); ion-selective electrode (I); nuclear magnetic resonance (N); theoretical calculation in gas phase or solution (Tgl); single-crystal X-ray analysis (X); conductivity (C); high-performance liquid chromatography (H); circular dichroism (CD). ^c^ ND–not determined.

**Table 2:** Comparisons of geometrical parameters and inclusion structures of four 2° amine TCAs embedded in the β-CD cavity, PRT (**1**), MPL (**2**), NRT (i), and DPM (ii) ^a^.

	PRT (1)	MPL (2)	NRT (i) ^b^	DPM (ii) ^c^
(1) Geometrical parameters				
(6-7/6-6)-Tricyclic core				
Butterfly angle *ε* (°) ^d^	131.4(4)	118.6(3)	116.7(3)	120.0(3)
Annellation angle *η* (°) ^e^	26.1(1)	2.5(3)	24.5(3)	28.9(3)
Twist angle *τ* (°) ^f^	–178.3(4)	1.7(3)	4.7(4)	–8.1(5)
C15–C10–C11–C12 torsion angle (°)	1.4(19)	–	44.1(16)	–53.7(12)
A-ring-centroid–B-ring-centroid distance *d*_AB_ (Å)	4.978	4.483	4.814	4.757
Side-chain at N5/C5				
C13–N5/C5–C16–C17 torsion angle (°)	59.1(10)	–167.4(7)	174.4(5)	–64.3(7)
N5/C5–C16–C17–C18 torsion angle (°)	179.3(8)	–172.1(10)	140.0(6)	–171.9(5)
N5’–A-ring centroid distance *d*_NA_ (Å)	5.928(9)	7.619(8)	7.441(6)	6.564(6)
N5’–B-ring centroid distance *d*_NB_ (Å)	7.543(8)	6.111(8)	6.055(6)	6.136(6)
*d*_NA_/*d*_NB_ ratio	0.786	1.247	1.229	1.070
(2) Inclusion structure				
Interplanar angle (°)				
A-ring vs. β-CD O4 plane	41.8(3)	85.4(2)	88.4(2)	83.7(2)
B-ring vs. β-CD O4 plane	89.7(2)	34.0(2)	25.1(1)	25.8(1)
Rings embedded in the β-CD cavity	B, C	A, C	A, C	A, C
Distance from TCA to β-CD (Å)				
A/B-ring centroid to O4 centroid (diagonal) ^g^	1.384	0.549	0.822	0.467
A/B-ring centroid to O4 plane (vertical)	1.384	0.551	0.822	0.464
C-ring centroid to O4 centroid	–1.286	–1.927	–1.819	–2.295
B/A-ring centroid to O4 centroid	–3.741	–4.127	–4.111	–4.475

^a^ All TCAs are in HCl form. ^b^ β-CD inclusion complexes with NRT [23]. ^c^ β-CD inclusion complexes with DPM [28]. ^d–f^ Interplanar angle of the A and B rings; Angle between C13–C12 and C14–C15; C13–C12–C15–C14 torsion angle (see atom numbering in Scheme 1). ^g^ When the β-CD O6-side points upwards, the positive (negative) values indicate that the TCA A/B/C-ring centroid is above (beneath) the β-CD O4-plane.

**Table 3:** Selected host–guest interactions in the β-CD–PRT and β-CD–MPL inclusion complexes deduced from X-ray analysis and DFT full-geometry optimization (Å, °).

Interaction ^a^	D–H	H···A	D···A	∠(DHA)	Interaction ^a^	D–H	H···A	D···A	∠(DHA)
X-ray					DFT ^e^				
β-CD–PRT HCl (1)					β-CD–PRT base				
N5’P–H1···O21 ^i,b^	0.89	2.41	3.087(10)	133.1	C52–H···*Cg*2 ^c^	1.10	3.75	4.80	162.2
N5’P–H1···O31 ^i^	0.89	2.06	2.856(11)	148.6	C55–H···*Cg*2	1.10	4.61	3.56	161.6
N5’P–H1···O21 ^i^	0.89	2.41	3.087(10)	133.1	C31–H···*Cg*1 ^c^	1.10	4.49	3.46	157.1
C51–H···*Cg*2 ^c^	0.98	3.69	4.461	137.8					
C55–H···*Cg*2	0.98	3.39	4.239	146.3					
C32–H···*Cg*1	0.98	3.21	4.135	158.9					
β-CD–MPL HCl (2)					β-CD–MPL base				
O61–H···N5’M ^ii,b^	0.82	2.44	3.159(12)	146.4	C51–H···*Cg*1 ^c^	1.10	3.93	4.98	160.7
N5’M–H1···O52 ^iii^	0.89	2.50	3.141(8)	129.2	C31–H···*Cg*2 ^c^	1.10	3.37	4.30	143.5
N5’M–H1···O62 ^iii^	0.89	2.10	2.930(11)	155.7					
N5’M–H2···O61 ^iii^	0.89	2.60	3.159(12)	121.7					
C51–H···*Cg*1 ^d^	0.98	3.577	4.515	161.1					
C55–H···*Cg*1	0.98	3.843	4.782	161.4					
C31–H···*Cg2*	0.98	3.018	3.903	150.9					
O21–H···*Cg2*	0.82	4.394	4.499	92.1					
O37–H···*Cg2*	0.82	3.930	4.520	132.0					

^a^ For full lists of intermolecular interactions, see Table S4, Table S5 (X-ray), and Table S6 (DFT). ^b^ Symmetry-related molecules with equivalent positions: ^(i)^ −*x* + 1, *y* − 0.5, −*z* + 2; ^(ii)^ −*x* + 1, *y* − 0.5, −*z* + 1.5; ^(iii)^ −*x* + 1, *y* + 0.5, −*z* + 1.5. ^c^ PRT aromatic centroids: *Cg*1 = A-ring (C1P−C2P−C3P−C4P−C13P−C12P), *Cg*2 = B-ring (C6P–C7P–C8P–C9P–C15P–C14P). ^d^ MPL aromatic centroids: *Cg*1 = A-ring (C1M−C2M−C3M−C4M−C13M−C12M), *Cg*2 = B-ring (C6M−C7M−C8M−C9M−C15M−C14M). ^e^ For stabilization and interaction energies, see Table S7.

## Data Availability

The data are contained within the article. Additional crystallographic and computational data are available in Supplementary Materials and the Cambridge Crystallographic Data Centre (CCDC).

## Supplementary Materials

The following are available online at https://www.mdpi.com/article/10.3390/ph14080812/s1, Table S1: Summary of the CD–3° amine TCA complexes characterized by various techniques, Table S2: X-ray single-crystal data collection and refinement statistics of **1** and **2**. Table S3: Selected geometrical parameters of two β-CD macrocycles of **1** and **2**, in comparison with those of β-CD–(–)-epicatechin and β-CD∙12H_2_O. Table S4: Hydrogen bond parameters and π···π interactions in β-CD–PRT HCl 0.4EtOH 12.9H_2_O (**1**), Table S5: Hydrogen bond parameters and π···π interactions in β-CD–MPL HCl 0.7EtOH 10.4H_2_O (**2**), Table S6: Hydrogen bond parameters in β-CD–PRT and β-CD–MPL inclusion complexes from DFT full-geometry optimization, Table S7: Stabilization and interaction energies of β-CD–PRT and β-CD–MPL, in comparison to other β-CD–TCA inclusion complexes from DFT full-geometry optimization, Table S8: Dispersion-corrected interaction energies of eight β-CD–TCA inclusion complexes from DFT/B97D calculations, Table S9: BSSE- and dispersion-corrected interaction energies of eight β-CD–TCA inclusion complexes from DFT/B97D calculations. Crystallographic data of **1** and **2** have been deposited at the Cambridge Crystallographic Data Centre (CCDC) under respective reference numbers 2093556 and 2093557. The author has read and agreed to the published version of the manuscript.
