# Supplementary material for: Distinctive Supramolecular Features of β-Cyclodextrin Inclusion Complexes with Antidepressants Protriptyline and Maprotiline: A Comprehensive Structural Investigation
Source: Pharmaceuticals (Basel). 2021 Aug 18;14(8):812. doi: 10.3390/ph14080812 (PMC8400697; doi:10.3390/ph14080812)
Supplement: Supplementary file 1 [file pharmaceuticals-14-00812-s001.zip › pharmaceuticals-1327581-supplementary.pdf]

Supplementary Materials

Distinctive Supramolecular Features of β-Cyclodextrin Inclusion Complexes with Antidepressants Protriptyline and Maprotiline: A Comprehensive Structural Investigation

Thammarat Aree

Department of Chemistry, Faculty of Science, Chulalongkorn University, Bangkok 10330, Thailand; athammar@chula.ac.th; Tel.: +66-2-2187584; Fax +66-2-2187598

Supplementary materials available:

1. Literature survey

Table S1. Summary of the CD–3° amine TCA complexes characterized by various techniques.

2. Crystallographic data

Table S2. X-ray single crystal data collection and refinement statistics of **1** and **2**.

Table S3. Selected geometrical parameters of two β-CD macrocycles of **1** and **2**, in comparison with those of β-CD–(–)-epicatechin and β-CD·12H_2O.

Table S4. Hydrogen bond parameters and π···π interactions in β-CD·PRT·HCl·0.4EtOH ·12.9H_2O (**1**).

Table S5. Hydrogen bond parameters and π···π interactions in β-CD·MPL·HCl·0.7EtOH ·10.4H_2O (**2**).

3. Computational data

Table S6. Hydrogen bond parameters in β-CD–PRT and β-CD–MPL inclusion complexes from DFT full-geometry optimization.

Table S7. Stabilization and interaction energies of β-CD–PRT and β-CD–MPL, in comparison to other β-CD–TCA inclusion complexes from DFT full-geometry optimization.

Table S8. Dispersion-corrected interaction energies of eight β-CD–TCA inclusion complexes from DFT/B97D calculations.

Table S9. BSSE- and dispersion-corrected interaction energies of eight β-CD–TCA inclusion complexes from DFT/B97D calculations.

References

1. Literature survey

Table S1. Summary of the CD–3° amine TCA complexes characterized by various techniques.

Host	Guest	Ratio	Inclusion mode [a]		K_a, M^{-1} (Tech.) [b]	Ref. [d]
			Aromatic	Side chain		
α-CD	AMT	1:1		✓	113 (FI)	[1]
α-CD	AMT	1:1		✓	0.06×10^{3} (I)	[2]
β-CD	AMT	1:1		✓	23.90×10^{3} (I)	[2]
β-CD	AMT	1:1		✓	3.19×10^{3} (C)	[3]
HP-β-CD	AMT	1:1	ND		1.03×10^{3} (C)	[4]
β-CD	AMT	1:1	✓ A/B		18.4×10^{3} (H)	[5]
β-CD	AMT	1:1	✓A/B		8.2×10^{3} (N)	[6]
β-CD	AMT	1:1	✓ A		ND (X)	[7]
β-CD	AMT	1:1	✓ A		ND (X)	[8]
β-CD	AMT	1:1		✓	ND (Tgl)	[8]
α-CD	IPM	1:1		✓	130 (FI)	[1]
α-CD	IPM	1:1		✓	0.08×10^{3} (I)	[2]
β-CD	IPM	1:1		✓	8.70×10^{3} (I)	[2]
β-CD	IPM	1:1		✓	1.50×10^{3} (C)	[3]
β-CD	IPM	1:1		✓	16.7 (U), 808 (F)	[9]
HP-β-CD	IPM	1:1	ND [c]		0.82×10^{3} (C)	[4]
β-CD	IPM	1:1	✓ A/B		8.8×10^{3} (H)	[5]
		1:1	✓	✓		
β-CD	IPM	1:2	✓ A/B		ND (N, L)	[10]
		2:1	✓ A+B			
β-CD	IPM	2:1	✓ A+B		ND (Tgl)	[10]
β-CD	IPM	1:1	✓ A		ND (Tg)	[11]
β-CD	IPM	1:1	✓ A		ND (X)	[12]
β-CD	IPM	1:1	✓ A	✓	ND (Tg)	[12]
β-CD	CPM	1:1	✓ A		9.42×10^{3} (U)	[13]
HP-β-CD	CPM	1:1	✓ A		9.58×10^{3} (U)	[13]
β-CD	CPM	1:1	✓ B		ND (X)	[14]
β-CD	CPM	1:1	✓ A+B		ND (Tg)	[14]
α-CD	DXP	1:1		✓	140 (FI)	[1]
α-CD	DXP	1:1		✓	0.05×10^{3} (I)	[2]
α-CD	DXP	1:1		✓	ND (Tg)	[15]
α-CD	DXP	2:1	✓ A+B		16.5×10^{3} (U), 19.6×10^{3} (F)	[15]
β-CD	DXP	1:1		✓	13.21×10^{3} (I)	[2]
β-CD	*E*-DXP	1:1		✓	36.0×10^{3} (E)	[16]
β-CD	*Z*-DXP	1:1		✓	22.7×10^{3} (E)	[16]
β-CD	DXP	3:1	✓ A+B	✓	ND (U, F)	[17]
β-CD	DXP	1:1	✓		397 (U), 624 (F)	[17]
β-CD	DXP	1:1	✓ A/B		13.4×10^{3} (H)	[5]
β-CD	DXP	1:1	✓ A+B		ND (N)	[18]
β-CD	DXP	1:1	✓ A		ND (Tg)	[11]
β-CD	DXP	2:1	✓ A+B		14.7×10^{3} (U), 16.2×10^{3} (F)	[15]
β-CD	DXP	1:1	✓ A+B		ND (X)	[14]
β-CD	DXP	1:1	✓ A+B		ND (Tg)	[14]

[a] TCA moiety included in CD cavity: aromatic rings A, B, or side chain.
[b] Binding constant (K_a) at 298 K derived from different techniques, mostly in solution: flow injection (FI); fluorescence (F); UV-vis (U); ion-selective electrode (I); capillary electrophoresis (E); light scattering (L); nuclear magnetic resonance (N); theoretical calculation in gas phase or solution (Tgl); single-crystal X-ray analysis (X); conductivity (C); high-performance liquid chromatography (H)
[c] ND – not determined.
[d] Full reference list is given on pages 11–12.

2. Crystallographic data

Table S2. X-ray single crystal data collection and refinement statistics of **1** and **2**.

	**1**<br>β-CD–Protriptyline HCl	**2**<br>β-CD–Maprotiline HCl
Abbreviated formula	β-CD·PRT·HCl ·0.4EtOH·12.9H_2O	β-CD·MPL·HCl ·0.7EtOH·10.4H_2O
Chemical formula	$(C_6H_{10}O_5)_7 \cdot C_{19}H_{21}N \cdot HCl \cdot 0.4(C_2H_6O) \cdot 12.9H_2O$	$(C_6H_{10}O_5)_7 \cdot C_{20}H_{23}N \cdot HCl \cdot 0.7(C_2H_6O) \cdot 10.4H_2O$
Formula weight	1670.92	1657.55
Crystal habit, color	Rod, colorless	Rod, colorless
Crystal size [mm]	0.42 × 0.40 × 0.38	0.46 × 0.42 × 0.30
Crystal system, space gr.	Monoclinic, $P2_1$ (No. 4)	Orthorhombic, $P2_12_12_1$ (No. 19)
a, b, c [Å]	15.6016(4), 18.9261(6), 16.4508(5)	14.9858(5), 18.6252(5), 29.9133(8)
α, β, γ [°]	90, 117.384(1), 90	90, 90, 90
V [Å^3]	4313.2(2)	8349.2(4)
Z	2	4
D_c [g cm^{-3}]	1.287	1.319
μ [mm^{-1}]	0.141	0.143
F(000)	1774	3526
Diffractometer	APEXII Kappa CCD (Bruker)	APEXII Kappa CCD (Bruker)
Wavelength [Å]	MoK_α, 0.71073	MoK_α, 0.71073
T [K]	296(2)	296(2)
Data collection	ω–ϕ scan, 0.4° step, 8 s expose	ω–ϕ scan, 0.5° step, 8 s expose
Frames collected	910	966
θ range [°]	1.82–25.38	1.87–25.35
Resolution [Å]	0.83	0.83
Completeness [%], R_{int}	99.9, 0.0354	99.8, 0.0284
Reflns collected / unique / observed	42205 / 15779 / 10114	53243 / 15279 / 11996
Data / restraints / parameters	15779, 5, 1007	15279, 29, 930
R_1 [a], wR_2 [$I > 2\sigma(I)$] [b]	0.0781, 0.2074	0.0768, 0.2127
R_1, wR_2 [all data], GoF	0.1181, 0.2443, 1.005	0.0948, 0.2329, 1.025
$\Delta\rho_{min}$, $\Delta\rho_{max}$ [e Å^{-3}]	–0.42, 0.56	–0.47, 0.69
CCDC number	2093556	2093557

[a,b] $R = \sum||F_o| - |F_c||/\sum|F_o|$; $wR = \sum\{w(F_o^2 - F_c^2)^2/\sum w(F_o^2)^2\}^{1/2}$.

Table S3. Selected geometrical parameters of two β-CD macrocycles of **1** and **2**, in comparison with those of β-CD–(–)-epicatechin and β-CD·12H_2O.

Residue	Puckering Q [Å] [a], θ [º] [b]				Tilt angle [º] [c]				O4 deviation [Å] [d]				O4(n)···O4(n – 1), O4(n)···centroid [Å]			
n	**1**	**2**	**β-CD–EC** [e]	**β-CD·12W** [f]	**1**	**2**	**β-CD–EC**	**β-CD·12W**	**1**	**2**	**β-CD–EC**	**β-CD·12W**	**1**	**2**	**β-CD–EC**	**β-CD·12W**
1	0.569(9)	0.569(6)	0.561(2)	0.570									4.498(7)	4.504(5)	4.403(2)	4.489
	4.7(9)	4.7(6)	6.7(2)	7.6	30.4(4)	13.6(2)	19.7(1)	15.0	0.128(4)	–0.191(3)	–0.180(1)	0.192	4.678(4)	4.944(3)	5.171	4.981
2	0.571(10)	0.557(6)	0.545(2)	0.583									4.445(7)	4.315(5)	4.263(2)	4.392
	1.5(10)	3.4(6)	5.6(2)	3.0	12.1(5)	18.8(1)	4.6(1)	26.2	–0.251(4)	–0.053(3)	–0.084(1)	0.091	5.119(4)	5.262(3)	5.397	5.153
3	0.557(9)	0.566(6)	0.574(2)	0.559									4.280(7)	4.303(5)	4.278(2)	4.286
	3.9(9)	2.9(6)	3.7(2)	3.9	5.6(3)	8.2(1)	6.9(1)	10.8	–0.071(4)	0.264(3)	0.325(1)	–0.195	5.282(4)	5.088(4)	4.581	5.122
4	0.562(11)	0.562(6)	0.557(2)	0.596									4.350(6)	4.442(5)	4.498(2)	4.443
	1.4(11)	4.0(6)	4.9(3)	1.4	12.3(5)	6.6(1)	33.7(1)	7.9	0.336(4)	–0.133(4)	–0.199(1)	–0.053	4.852(4)	4.735(4)	4.827	4.856
5	0.577(8)	0.558(8)	0.578(2)	0.579									4.419(6)	4.468(6)	4.322(2)	4.452
	3.4(8)	2.3(8)	3.2(2)	2.0	28.7(4)	25.5(3)	1.9(1)	10.7	–0.117(4)	–0.136(4)	–0.073(1)	0.276	4.715(4)	5.130(3)	5.435	5.054
6	0.552(8)	0.537(6)	0.565(2)	0.571									4.359(6)	4.203(6)	4.209(2)	4.247
	2.7(8)	7.9(6)	4.9(2)	3.9	5.6(4)	11.2(2)	14.6(1)	20.3	–0.279(4)	0.128(3)	0.087(1)	–0.115	5.311(4)	5.286(4)	5.075	5.184
7	0.561(8)	0.557(6)	0.582(2)	0.567									4.171(6)	4.405(5)	4.626(2)	4.338
	6.9(9)	0.0(6)	8.8(2)	3.7	12.1(3)	0.4(1)	30.6(1)	6.4	0.253(4)	0.122(3)	0.124(1)	–0.196	5.091(4)	4.797(4)	4.632	4.913
													0.327 [g]	*0.301*	*0.417*	*0.242*
													0.633 [g]	*0.551*	*0.854*	*0.328*
													0.873 [g]	*0.871*	*0.876*	*0.870*

[a,b] A perfect cyclohexane chair (for R(C–C) = 1.54 Å) has puckering amplitude Q = 0.63 Å and angle describing the polar position θ = 0º [19].

[c] Interplanar angle of the plane through C1(n), C4(n), O4(n) and O4(n – 1) against the O4 plane.

[d] Deviation of glycosidic O4 atoms from the least-squares plane through the seven O4 atoms.

[e] β-CD–(–)-epicatechin (EC) [20].

[f] β-CD·12H_2O [21].

[g] Ranges of the O4(n)···O4(n – 1), O4(n)···centroid distances and the average of their ratios are in *italics*; for an ideal heptagon, the ratio is 0.868.

[h] Endocyclic torsion angles ϕ and ψ at glycosidic O4, defined as O5(n + 1)–C1(n + 1)–O4(n)–C4(n) and C1(n + 1)–O4(n)–C4(n)–C5(n), respectively.

[i] Averages of ϕ and ψ are in *italics*; for the β-CD roundness, the sum of averages should be zero [22].

[j] Exocyclic torsion angles χ and ω are defined as C4–C5–C6–O6 and O5–C5–C6–O6, respectively.

[k,m] Doubly disordered O64–H group with occupancy factors 0.77 and 0.23 for respective sites A and B.

Table S3. Continued.

Residue	O3(n)···O2(n + 1) distance [Å]				Torsion angles ϕ [h], ψ [h] [°]				Torsion angles χ [j], ω [j] [°]			
n	**1**	**2**	**β-CD–EC**	**β-CD·12W**	**1**	**2**	**β-CD–EC**	**β-CD·12W**	**1**	**2**	**β-CD–EC**	**β-CD·12W**
1	2.866(10)	2.811(7)	2.924(2)	2.957	116.3(7)	114.1(5)	100.7(2)	119.3	59.5(11)	48.5(9)	53.5(2)	–169.4
					–100.4(6)	–105.3(5)	–121.5(2)	–95.9	–60.8(10)	–71.4(7)	–66.8(2)	70.5
2	2.918(9)	2.816(7)	2.765(2)	2.875	103.1(7)	112.2(5)	111.6(2)	110.5	54.6(10)	179.8(6)	58.7(3)	–173.9
					–123.3(7)	–112.9(5)	–118.1(2)	–106.6	–65.9(9)	60.2(8)	–62.7(2)	71.0
3	2.890(10)	2.888(7)	2.762(2)	2.902	104.3(7)	107.7(5)	129.1(2)	102.5	58.4(9)	56.2(7)	48.9(2)	58.7
					–114.4(6)	–118.6(5)	–97.4(2)	–121.1	–64.4(10)	–65.4(7)	–72.5(2)	–60.8
4	2.853(7)	2.898(9)	3.346(3)	2.783	109.7(8)	105.7(5)	90.1(2)	107.7	47.7(15) [k] 77.7(49) [m]	55.2(7)	55.7(3)	57.0
					–110.3(8)	–112.4(5)	–131.9(2)	–109.4	–68.9(13) [k] –60.7(50) [m]	–64.6(7)	–64.5(2)	–61.0
5	2.851(8)	2.817(7)	2.828(3)	2.770	119.4(6)	118.5(6)	117.1(2)	110.7	56.1(9)	65.0(9)	60.7(2)	50.7
					–101.7(7)	–100.6(7)	–105.9(2)	–114.1	–63.5(8)	–56.6(8)	–60.9(2)	–71.0
6	2.926(9)	2.844(7)	3.246(3)	2.855	99.1(6)	104.9(6)	105.9(2)	120.0	56.9(7)	–175.8(7)	–167.8(2)	–175.4
					–134.9(5)	–119.5(6)	–96.5(2)	–109.8	–66.2(7)	62.9(10)	69.8(2)	64.7
7	2.919(9)	2.829(7)	2.833(3)	2.862	111.3(6)	108.3(6)	102.7(2)	103.0	–169.2(6)	56.2(7)	54.2(3)	52.1
					–103.8(6)	–118.5(6)	–118.4(2)	–125.7	68.7(8)	–65.1(6)	–64.7(3)	–62.9
					109.0 [i]	*110.2*	*108.2*	*110.5*				
					–112.7 [i]	*–112.5*	*–112.8*	*–111.8*				
					–3.7 [i]	*–2.3*	*–4.6*	*–1.3*				

[a,b] A perfect cyclohexane chair (for R(C–C) = 1.54 Å) has puckering amplitude Q = 0.63 Å and angle describing the polar position $\theta = 0^{\circ}$ [19].
[c] Interplanar angle of the plane through C1(n), C4(n), O4(n) and O4(n – 1) against the O4 plane.
[d] Deviation of glycosidic O4 atoms from the least-squares plane through the seven O4 atoms.
[e] β-CD–(–)-epicatechin (EC) [20].
[f] β-CD·12H_2O [21].
[g] Ranges of the O4(n)···O4(n – 1), O4(n)···centroid distances and the average of their ratios are in *italics*; for an ideal heptagon, the ratio is 0.868.
[h] Endocyclic torsion angles ϕ and ψ at glycosidic O4, defined as O5(n + 1)–C1(n + 1)–O4(n)–C4(n) and C1(n + 1)–O4(n)–C4(n)–C5(n), respectively.
[i] Averages of ϕ and ψ are in *italics*; for the β-CD roundness, the sum of averages should be zero [22].
[j] Exocyclic torsion angles χ and ω are defined as C4–C5–C6–O6 and O5–C5–C6–O6, respectively.
[k,m] Doubly disordered O64–H group with occupancy factors 0.77 and 0.23 for respective sites A and B.

Table S4. (a) Hydrogen bond parameters in β-CD·PRT·HCl·0.4EtOH·12.9H_2O (**1**) [Å, °].

D–H···A	D–H	H···A	D···A	∠(DHA)	D–H···A	D–H	H···A	D···A	∠(DHA)
β-*CD*–β-*CD*					O26–H···O3W	0.82	2.42	3.064(14)	135.8
O21–H···O37	0.82	2.13	2.919(9)	162.4	O36–H···O11WA	0.82	2.53	3.28(4)	151.6
O31–H···O22	0.82	2.11	2.866(10)	153.1	O36–H···O11WB	0.82	1.94	2.71(4)	155.1
O32–H···O23	0.82	2.15	2.918(9)	156.5	O37–H···O8W	0.82	1.97	2.77(3)	166.1
O24–H···O33	0.82	2.18	2.890(10)	145.0	O1W–H2···O67 [iii]	0.96	2.01	2.664(14)	123.1
O34–H···O25	0.82	2.18	2.853(7)	139.6	O3W–H1···O33 [vi]	0.97	2.55	3.091(14)	115.6
O64A–H···O61 [iii b c]	0.82	2.04	2.747(19)	144.7	*PRT*–β-*CD/Cl*				
O25–H···O62 [iii]	0.82	2.08	2.728(8)	135.9	N5'P–H1···O21 [viii]	0.89	2.41	3.087(10)	133.1
O35–H···O26	0.82	2.04	2.851(8)	171.1	N5'P–H1···O31 [viii]	0.89	2.06	2.856(11)	148.6
O66–H···O63 [vi]	0.82	2.21	2.878(9)	138.9	N5'P–H2···Cl2	0.89	2.07	2.934(15)	162.3
O27–H···O36	0.82	2.35	2.926(8)	128.3	C51–H···*Cg*2 [d]	0.98	3.69	4.461	137.8
O67–H···O35 [vii]	0.82	2.06	2.733(9)	138.9	C55–H···*Cg*2	0.98	3.39	4.239	146.3
β-*CD*–*H₂O/EtOH/Cl* [a]					C32–H···*Cg*1	0.98	3.21	4.135	158.9
O61–H···O12WB [i]	0.82	1.69	2.48(4)	161.5	*H_2O–H_2O*				
O22–H···Cl1	0.82	2.41	3.097(11)	142.0	O1E–H···O7W	0.82	2.14	2.70(5)	125.1
O62–H···O7W	0.82	1.90	2.67(2)	155.6	O1W–H1···O12WA	0.96	1.98	2.93(3)	172.9
O23–H···O10WA	0.82	2.05	2.85(3)	163.5	O2W–H1···O6W [vii]	0.96	1.99	2.70(3)	129.1
O23–H···O10WB	0.82	2.25	2.82(4)	127.2	O3W–H2···O1W [vi]	0.98	2.39	3.00(3)	120.3
O33–H···O3W [ii]	0.82	2.43	3.091(14)	138.2	O4W–H1···O2W [vii]	0.96	2.35	2.83(3)	110.4
O63–H···O12WA	0.82	2.33	2.74(2)	112.4	O4W–H2···O7W [vi]	0.96	1.62	2.53(4)	157.1
O64B–H···O8W [iv]	0.82	2.62	3.16(6)	124.7	O5W–H1···O1E [ix]	0.96	2.23	2.99(5)	135.5
O65–H···Cl1 [v]	0.82	2.26	3.073(12)	174.0	O6W–H1···O12WA [iii]	0.96	1.84	2.72(2)	151.9
O26–H···Cl1 [viii]	0.82	2.78	3.403(12)	133.7					

[a] Site occupancy factors (SOFs) are as follows:
12.9 water molecules are distributed over 20 sites: 1.0 (O1W, O3W, O5W, O6W); 0.8 (O2W, O4W); 0.7 (O7W, O8W, O10WA, O12WA); 0.6 (O9WA); 0.5 (O10WB, O11WA/B, O12WB/C); 0.4 (O9WB, O14W); 0.3 (O13W, O15W)
EtOH: 0.4 (O1E); one twofold disordered chloride: 0.5 (Cl1, Cl2)

[b] Equivalent positions: (i) –*x*, *y* + 0.5, –*z* + 1; (ii) *x* – 1, *y*, *z*; (iii) –*x*, *y* – 0.5, –*z* + 1; (iv) *x* – 1, *y*, *z* – 1; (v) *x*, *y*, *z* – 1; (vi) *x* + 1, *y*, *z*; (vii) –*x* + 1, *y* + 0.5, –*z* + 1; (viii) –*x* + 1, *y* – 0.5, –*z* + 2; (ix) –*x*, *y* – 0.5, –*z* + 2.

[c] Twofold disordered O64–H group with occupancy factors 0.77 and 0.23 for respective sites A and B.

[d] *Cg*1 = A-ring (C1P–C2P–C3P–C4P–C13P–C12P), *Cg*2 = B-ring (C6P–C7P–C8P–C9P–C15P–C14P)

(b) π···π interactions in β-CD·PRT·HCl·0.4EtOH·12.9H_2O (**1**) [Å, °].

There is no π···π interaction in **1**.

Table S5. (a) Hydrogen bond parameters in β-CD·MPL·HCl·0.7EtOH·10.4H_2O (**2**) [Å, °].

D–H···A	D–H	H···A	D···A	∠(DHA)	D–H···A	D–H	H···A	D···A	∠(DHA)
β-*CD*–β-*CD*									
O22–H···O31	0.82	2.11	2.811(7)	143.4	O3W–H2···O66	0.96	2.06	2.888(11)	143.2
O32–H···O23	0.82	2.00	2.816(7)	171.8	O4W–H2···O37	0.96	1.89	2.832(7)	166.8
O24–H···O33	0.82	2.08	2.888(7)	166.2	O67–H···Cl2	0.82	2.04	2.833(9)	163.7
O64–H···O67 [i b c]	0.82	2.15	2.945(8)	164.8	*MPL*–β-*CD*/H_2O				
O25–H···O34	0.82	2.10	2.898(9)	164.2	O61–H···N5'M [ii]	0.82	2.44	3.159(12)	146.4
O66–H···O32 [ii]	0.82	2.04	2.805(8)	154.2	N5'M–H1···O52 [iv]	0.89	2.50	3.141(8)	129.2
O26–H···O35	0.82	2.01	2.817(7)	167.7	N5'M–H1···O62 [iv]	0.89	2.10	2.930(11)	155.7
O27–H···O36	0.82	2.23	2.844(7)	132.4	N5'M–H2···O61 [iv]	0.89	2.60	3.159(12)	121.7
O37–H···O21	0.82	2.17	2.829(7)	137.6	N5'M–H2···O13W	0.89	2.33	3.09(6)	143.3
β-*CD*–H_2O/*EtOH*/*Cl* [a]					C51–H···*Cg*1 [c]	0.98	3.577	4.515	161.1
O21–H···O6WA	0.82	2.12	2.825(11)	144.6	C55–H···*Cg*1	0.98	3.843	4.782	161.4
O21–H···O6WB	0.82	2.09	2.90(2)	170.5	C31–H···*Cg2*	0.98	3.018	3.903	150.9
O31–H···O6WB	0.82	2.29	3.07(2)	159.5	O21–H···*Cg2*	0.82	4.394	4.499	92.1
O1E–H···O22 [ii]	0.82	2.07	2.81(2)	150.2	O37–H···*Cg2*	0.82	3.930	4.520	132.0
O62–H···O4W [ii]	0.82	1.92	2.743(9)	175.3	H_2O–H_2O/*EtOH*	0.96	1.88	2.81(2)	161.7
O23–H···Cl1	0.82	1.96	2.768(7)	170.5	O1W–H1···O11W [ii]	0.96	2.11	2.96(2)	146.3
O33–H···O6WA [i]	0.82	2.22	2.972(12)	152.3	O1W–H2···O12W	0.96	2.36	3.32(4)	172.2
O63–H···Cl2 [i]	0.82	2.03	2.846(7)	170.5	O2W–H1···O6WA [ii]	0.96	1.90	2.785(13)	151.5
O5W–H2···O24 [iii]	0.96	2.20	2.872(11)	126.2	O2W–H1···O6WB [ii]	0.96	2.26	3.02(2)	136.2
O34–H···O1W [iv]	0.82	2.52	3.120(15)	131.0	O3W–H1···O1E	0.96	1.88	2.75(2)	149.1
O35–H···O11W	0.82	2.06	2.87(2)	175.6	O4W–H1···O10W	0.96	2.04	2.79(2)	134.2
O65–H···O3W	0.82	2.23	2.824(12)	129.8	O5W–H1···O4W	0.96	2.06	2.784(13)	131.0
O2W–H2···O65	0.96	2.19	2.859(11)	125.5					

[a] Site occupancy factors (SOFs) are as follows:
10.4 water molecules distributed over 16 sites: 1.0 (O1W–O5W); 0.8 (O7W); 0.7 (O6WA, O8WA);
0.6 (O10W, O11W); 0.4 (O9W, O12W), 0.3 (O6WB, O8WB, O13W, O14W);
EtOH: 0.7 (O1E); one twofold disordered chloride: 0.5 (Cl1, Cl2)

[b] Equivalent positions: (i) $x + 1, y, z$; (ii) $-x + 1, y - 0.5, -z + 1.5$; (iii) $x - 1, y, z$; (iv) $-x + 1, y + 0.5, -z + 1.5$.

[c] *Cg*1 = A-ring (C1M–C2M–C3M–C4M–C13M–C12M), *Cg*2 = B-ring (C6M–C7M–C8M–C9M–C15M–C14M)

(b) π···π interactions in β-CD·MPL·HCl·0.7EtOH·10.4H_2O (**2**) [Å, °].

Cg(*I*)	*Cg*(*J*)	*Cg*–*Cg*	Alpha	*CgI*_Perp	*CgJ*_Perp	Type
*Cg*2	*Cg*1	4.482(3)	61.4(3)	2.241(2)	2.331(2)	Edge-to-face
*Cg*2	*Cg*1($-x + 1, y + 0.5, -z + 1.5$)	5.587(3)	60.4(3)	1.280(2)	4.076(2)	Edge-to-face

Note:
- *Cg*(*I*) = Plane number *I*
  *Cg*1 = A-ring (C1M–C2M–C3M–C4M–C13M–C12M), *Cg*2 = B-ring (C6M–C7M–C8M–C9M–C15M–C14M)
- Alpha = Interplanar angle between planes *I* and *J* [°]
- *Cg*–*Cg* = Distance between ring centroids [Å]
- *CgI*_Perp = Perpendicular distance of *Cg*(*I*) on ring *J* [Å]
- *CgJ*_Perp = Perpendicular distance of *Cg*(*J*) on ring *I* [Å]

3. Computational data

Table S6. Hydrogen bond parameters in β-CD–PRT and β-CD–MPL inclusion complexes from DFT full-geometry optimization [Å, °].[a]

D–H···A	D–H	H···A	D···A	∠(DHA)	D–H···A	D–H	H···A	D···A	∠(DHA)
β-CD·PRT [b]			β–CD	conformation					
β-*CD*–β-*CD*			Distorted	round	β-*CD*–*PRT*				
O21–H···O37	0.98	2.09	3.00	155.8	C52–H···*Cg*2 [c]	1.10	3.75	4.80	162.2
O31–H···O22	0.98	1.96	2.91	163.5	C55–H···*Cg*2	1.10	4.61	3.56	161.6
O32–H···O23	0.98	1.90	2.86	165.8	C31–H···*Cg*1 [c]	1.10	4.49	3.46	157.1
O24–H···O33	0.98	2.11	2.95	143.6					
O34–H···O25	0.98	1.95	2.91	167.0					
O35–H···O26	0.98	1.91	2.87	167.2					
O27–H···O36	0.98	1.95	2.92	167.0					
β-CD·MPL [b]									
β-*CD*–β-*CD*			Distorted	round	β-*CD*–*MPL*				
O21–H···O37	0.98	2.05	2.98	158.3	C51–H···*Cg*1 [c]	1.10	3.93	4.98	160.7
O61–H···O52	0.98	2.03	2.93	151.4	C31–H···*Cg*2 [c]	1.10	3.37	4.30	143.5
O22–H···O31	0.98	1.95	2.90	160.6					
O32–H···O23	0.98	1.91	2.88	167.0					
O24–H···O33	0.98	2.01	2.90	151.2					
O25–H···O34	0.98	1.98	2.93	163.5					
O65–H···O56	0.98	2.12	2.97	144.5					
O26–H···.O35	0.98	1.98	2.94	165.9					
O27–H···O36	0.98	2.05	3.00	162.5					
O67–H···O51	0.98	1.99	2.90	153.8					

[a] DFT energy minimization in vacuum at the B3LYP/6–31+G(d)/4–31G level, see also Figure 8 and Table 3.
[b] X-ray-derived structures are used as starting models.
[c] C/O–H···π interactions with *Cg*1 and *Cg2* as the centroids of A-ring (C1–C2–C3–C4–C13–C12) and B-ring (C6–C7–C8–C9–C15–C14), respectively.

Table S7. Stabilization and interaction energies of β-CD–PRT and β-CD–MPL, in comparison to other β-CD–TCA inclusion complexes from DFT full-geometry optimization. [a]

	β-CD–PRT	β-CD–MPL	β-CD–NRT [f]	β-CD–DPM [g]	β-CD–AMT [f]	β-CD–IPM [g]	β-CD–CPM [h]	β-CD–*E*-DXP [h]	β-CD–*Z*-DXP [h]
E_{cpx} [b]	−5063.43953	−5102.71021	−5063.43343	−5080.72090	−5102.68430	−5119.98451	−5579.58468	−5138.63232	−5138.63454
$E_{β\text{-CD\_opt}}$ [c]	−4272.96510	−4272.96490	−4272.96876	−4272.96328	−4272.96532	−4272.96689	−4272.98021	−4272.96876	−4272.97420
E_{D_opt}	−790.46387	−829.73394	−790.45569	−807.75084	−829.71091	−847.00646	−1306.59775	−865.65107	−865.64700
$E_{β\text{-CD\_sp}}$	−4272.96318	−4272.96412	−4272.96673	−4272.96081	−4272.96295	−4272.96524	−4272.97744	−4272.96787	−4272.97340
E_{D_sp}	−790.46371	−829.73380	−790.45534	−807.74992	−829.71070	−847.00618	−1306.59752	−865.64185	−865.63828
ΔE_{stb} [Hartree] [d]	−0.01055	−0.01137	−0.00898	−0.00678	−0.00807	−0.01115	−0.00673	−0.01249	−0.01334
ΔE_{stb} [kcal mol^{-1}]	−6.62	−7.13	−5.64	−4.25	−5.06	−7.00	−4.22	−7.84	−8.37
ΔE_{int} [Hartree] [e]	−0.01264	−0.01228	−0.01136	−0.01017	−0.01065	−0.01309	−0.00972	−0.02261	−0.02287
ΔE_{int} [kcal mol^{-1}]	−7.93	−7.71	−7.13	−6.38	−6.68	−8.21	−6.10	−14.19	−14.35
Host-guest interactions	C–H···π	C–H···π	C–H···π	C–H···π	C–H···π	C–H···π	C–H···π	C–H···π	C–H···π

[a] DFT/B3LYP calculations in the gas phase with mixed basis sets 4−31G for C atoms and 6−31+G* for H, N, O, F, Cl atoms were carried out using program GAUSSIAN09 [23]. X-ray-derived structures were used as starting models, see also Figure 8 and Table S6.

[b] Original unit of *E* is Hartree [1 H = 627.5 kcal mol^{-1}].

[c] $E_{β\text{-CD\_opt}}$ in vacuum of the uncomplexed β-CD·12H_2O [21] is −4272.96662 H.

[d,e] Stabilization energy, $\Delta E_{stb} = E_{cpx} - (E_{β\text{-CD\_opt}} + E_{D\_opt})$

Interaction energy, $\Delta E_{int} = E_{cpx} - (E_{β\text{-CD\_sp}} + E_{D\_sp})$,

where E_{cpx}, $E_{β\text{-CD\_opt}}$ and E_{D_opt} are the molecular energies from full optimization of complex, host β-CD and drug TCA in freebase form, respectively;

$E_{β\text{-CD\_sp}}$ and E_{D_sp} are the corresponding single-point energies in the complexed states.

[f] β-CD inclusion complexes with NRT and AMT [8].

[g] β-CD inclusion complexes with DPM and IPM [12].

[h] β-CD inclusion complexes with CPM and *E*/*Z*-DXP [14].

Table S8. Dispersion-corrected interaction energies of eight β-CD–TCA inclusion complexes from DFT/B97D calculations. [a]

	β-CD–PRT	β-CD–MPL	β-CD–NRT	β-CD–DPM	β-CD–AMT	β-CD–IPM	β-CD–CPM	β-CD–*E*-DXP	β-CD–*Z*-DXP	β-CD–*E*-DXP [d]	β-CD–*E*-DXP [e]
E_{cpx} [b]	−5060.33917	−5099.58250	−5060.33092	−5077.61098	−5099.55712	−5116.84912	−5576.47218	−5135.48644	−5135.49822	−5138.76012	−5140.29332
$E_{β\text{-CD\_sp}}$	−4270.36980	−4270.37381	−4270.37339	−4270.36722	−4270.36849	−4270.37598	−4270.38452	−4270.37939	−4270.38342	−4272.69870	−4274.02846
E_{D_sp}	−789.90609	−829.15065	−789.89585	−807.18526	−829.12724	−846.41818	−1306.02383	−865.05040	−865.04513	−866.00522	−866.21921
ΔE_{int} [Hartree] [c]	−0.06328	−0.05804	−0.06168	−0.05850	−0.06139	−0.05495	−0.06383	−0.05665	−0.06967	−0.05620	−0.04565
ΔE_{int} [kcal mol^{-1}]	−39.71	−36.42	−38.71	−36.71	−38.52	−34.48	−40.05	−35.55	−43.72	−35.26	−28.65

[a] DFT/B97D calculations in the gas phase with mixed basis sets 4−31G for C atoms and 6−31+G* for H, N, O, F, Cl atoms were carried out using program GAUSSIAN09 [23]. DFT/B3LYP-optimized structures were used for single-point energy calculations, see also Table S7.
[b] Original unit of *E* is Hartree [1 H = 627.5 kcal mol^{-1}].
[c] Interaction energy, $\Delta E_{int} = E_{cpx} - (E_{β\text{-CD\_sp}} + E_{D\_sp})$, where E_{cpx}, $E_{β\text{-CD\_sp}}$ and E_{D_sp} are the single-point energies of complex, host β-CD and drug TCA in freebase form, respectively.
[d,e] Different basis sets 6-31G(d,p) and 6-311++G(2d,p) were used for single-point energy calculations of the β-CD–*E*-DXP complex.

Table S9. BSSE- and dispersion-corrected interaction energies of eight β-CD–TCA inclusion complexes from DFT/B97D calculations. [a]

	β-CD–PRT	β-CD–MPL	β-CD–NRT	β-CD–DPM	β-CD–AMT	β-CD–IPM	β-CD–CPM	β-CD–*E*-DXP	β-CD–*Z*-DXP
E_{cpx_BSSE} [b]	−5060.32159	−5099.56676	−5060.31412	−5077.59455	−5099.53961	−5116.83408	−5576.45497	−5135.47075	−5135.47897
$E_{β\text{-CD(cpx)\_sp}}$	−4270.37323	−4270.37701	−4270.37705	−4270.37091	−4270.37180	−4270.37919	−4270.38848	−4270.38283	−4270.38765
$E_{D(cpx)\_sp}$	−789.92015	−829.16331	−789.90927	−807.19824	−829.14144	−846.43028	−1306.03692	−865.06269	−865.06016
ΔE_{BSSE} [Hartree] [c]	0.01758	0.01574	0.01681	0.01643	0.01751	0.01504	0.01721	0.01569	0.01926
ΔE_{BSSE} [kcal mol^{-1}]	11.03	9.88	10.55	10.31	10.99	9.44	10.80	9.85	12.08
ΔE_{int_BSSE} [Hartree] [d]	−0.04579	−0.04218	−0.04460	−0.04184	−0.04388	−0.03965	−0.04678	−0.04092	−0.05041
ΔE_{int_BSSE} [kcal mol^{-1}]	−28.73	−26.47	−27.99	−26.25	−27.54	−24.88	−29.35	−25.68	−31.63

[a] DFT/B97D calculations in the gas phase with mixed basis sets 4−31G for C atoms and 6−31+G* for H, N, O, F, Cl atoms were carried out using program GAUSSIAN09 [23]. DFT/B3LYP-optimized structures were used for single-point energy calculations, see also Tables S7 and S8.
[b] Original unit of *E* is Hartree [1 H = 627.5 kcal mol^{-1}].
[c] The basis set superposition error (BSSE) energy by counterpoise correction [24], $\Delta E_{BSSE} = E_{cpx\_BSSE} - E_{cpx} = [E_{β\text{-CD\_sp}} - E_{β\text{-CD(cpx)\_sp}}] + [E_{D\_sp} - E_{D(cpx)\_sp}]$ where E_{cpx_BSSE} and E_{cpx} are corrected and uncorrected complex energies, respectively.
[d] BSSE-corrected interaction energy, $\Delta E_{int\_BSSE} = E_{cpx} - (E_{β\text{-CD(cpx)\_sp}} + E_{D(cpx)\_sp}) = \Delta E_{int} + \Delta E_{BSSE}$ where $E_{β\text{-CD(cpx)\_sp}}$ and $E_{D(cpx)\_sp}$ are energies of two components in the complex.

References

1. Georgiou, M.E.; Koupparis, M.A.; Georgiou, C.A. Rapid automated spectrophotometric competitive complexation studies of drugs with cyclodextrins using the flow injection gradient technique: tricyclic antidepressant drugs with α-cyclodextrin. *Analyst* **1999**, *124*, 391–396. https://doi.org/10.1039/A808426B.
2. Valsami, G.N.; Koupparis, M.A.; Macheras, P.E. Complexation studies of cyclodextrins with tricyclic antidepressants using ion-selective electrodes. *Pharm. Res.* **1992**, *9*, 94–100. https://doi.org/10.1023/A:1018940013006.
3. Junquera, E.; Romero, J.C.; Aicart, E. Behavior of tricyclic antidepressants in aqueous solution: self-aggregation and association with β-cyclodextrin. *Langmuir* **2001**, *17*, 1826–1832. https://doi.org/10.1021/la000819q.
4. Cano, J.; Rodriguez, A.; Aicart, E.; Junquera, E. Temperature effect on the complex formation between tricyclic antidepressant drugs (amitriptyline or imipramine) and hydroxypropyl-β-cyclodextrin in water. *J. Incl. Phenom. Macrocycl. Chem.* **2007**, *59*, 279–285. https://doi.org/10.1007/s10847-007-9328-x.
5. Piperaki, S.; Parissi-Poulou, M.; Koupparis, M. A separation study of tricyclic antidepressant drugs by HPLC with β-cyclodextrin bonded stationary phase. *J. Liq. Chromatogr. Relat. Technol.* **1993**, *16*, 3487–3508. https://doi.org/10.1080/10826079308019703.
6. Di Pietro, M.E.; Ferro, M.; Mele, A. Inclusion complexes of tricyclic drugs and β-cyclodextrin: Inherent chirality and dynamic behaviour. *Int. J. Pharm.* **2020**, *588*, 119775. https://doi.org/10.1016/j.ijpharm.2020.119775.
7. Castiglione, F.; Ganazzoli, F.; Malpezzi, L.; Mele, A.; Panzeri, W.; Raffaini, G. Inclusion complexes of β-cyclodextrin with tricyclic drugs: an X-ray diffraction, NMR and molecular dynamics study. *Beilstein J. Org. Chem.* **2017**, *13*, 714–719. https://doi.org/10.3762/bjoc.13.70.
8. Aree, T. β-Cyclodextrin encapsulation of nortriptyline HCl and amitriptyline HCl: molecular insights from single-crystal X-ray diffraction and DFT calculation. *Int. J. Pharm.* **2020**, *575*, 118899. https://doi.org/10.1016/j.ijpharm.2019.118899.
9. Viswalingam, M.; Prabu, S.; Sivakumar, K.; Rajamohan, R. Preparation and characterization of a imipramine–β-cyclodextrin inclusion complex. *Instrum. Sci. Technol.* **2016**, *44*, 651–671. https://doi.org/10.1080/10739149.2016.1177726.
10. Sousa, F.B.D.; Denadai, A.M.L.; Lula, I.S.; Nascimento Jr, C.S.; Neto, N.S.F.; Lima, A.C.; ... Sinisterra, R.D. Supramolecular self-assembly of cyclodextrin and higher water soluble guest: Thermodynamics and topological studies. *J. Am. Chem. Soc.* **2008**, *130*, 8426–8436. https://doi.org/10.1021/ja801080v.
11. Jenita, M.J.; Prabhu, A.; Rajendiran, N. Theoretical study of inclusion complexation of tricyclic antidepressant drugs with β-cyclodextrin. *Ind. J. Chem.* **2012**, *51A*, 1686–1694. http://nopr.niscair.res.in/handle/123456789/15201.
12. Aree, T. β-Cyclodextrin inclusion complexation with tricyclic antidepressants desipramine and imipramine: a structural chemistry perspective. *J. Pharm. Sci.* **2020**, *109*, 3086–3094. https://doi.org/10.1016/j.xphs.2020.07.007.
13. Misiuk, W.; Zalewska, M. Study on the inclusion interactions of β-cyclodextrin and its derivative with clomipramine by spectroscopy and its analytic application. *Anal. Lett.* **2008**, *41*, 543–560. https://doi.org/10.1080/00032710801910650.
14. Aree, T. Supramolecular Complexes of β-Cyclodextrin with Clomipramine and Doxepin: Effect of the Ring Substituent and Component of Drugs on Their Inclusion Topologies and Structural Flexibilities. *Pharmaceuticals* **2020**, *13*, 278. https://doi.org/10.3390/ph13100278.

15. Rajendiran, N.; Sankaranarayanan, R.K.; Saravanan, J. A study of supramolecular host–guest interaction of dothiepin and doxepin drugs with cyclodextrin macrocycles. *J. Mol. Struct.* **2014**, *1067*, 252–260. https://doi.org/10.1016/j.molstruc.2014.03.051.
16. Jones, C.J.; Larive, C.K. Microcoil NMR study of the interactions between doxepin, β-cyclodextrin, and acetate during capillary isotachophoresis. *Anal. Chem.* **2012**, *84*, 7099–7106. https://doi.org/10.1021/ac301401p.
17. Sankaranarayanan, R.K.; Siva, S.; Venkatesh, G.; Prabhu, A.A.M.; Rajendiran, N. Dual fluorescence of dothiepin, doxepin drugs—effect of solvents and β-cyclodextrin. *J. Mol. Liq.* **2011**, *161*, 107–114. https://doi.org/10.1016/j.molliq.2011.04.016.
18. Cruz, J.R.; Becker, B.A.; Morris, K.F.; Larive, C.K. NMR characterization of the host–guest inclusion complex between β-cyclodextrin and doxepin. *Magn. Reson. Chem*. **2008**, *46*, 838–845. https://doi.org/10.1002/mrc.2267.
19. Cremer, D.T.; Pople, J.A. General definition of ring puckering coordinates. *J. Am. Chem. Soc.* **1975**, *97*, 1354-1358. https://doi.org/10.1021/ja00839a011.
20. Aree, T.; Jongrungruangchok, S. Crystallographic evidence for β-cyclodextrin inclusion complexation facilitating the improvement of antioxidant activity of tea (+)-catechin and (−)-epicatechin. *Carbohydr. Polym.* **2016**, *140*, 362–373. https://doi.org/10.1016/j.carbpol.2015.12.066.
21. Lindner, K.; Saenger, W. Crystal and molecular structure of cyclohepta-amylose dodecahydrate. *Carbohydr. Res.* **1982**, *99*, 103–115. https://doi.org/10.1016/S0008-6215(00)81901-1.
22. French, A.D.; Johnson, G.P. Linkage and pyranosyl ring twisting in cyclodextrins. *Carbohydr. Res.* **2007**, 342, 1223–1237. https://doi.org/10.1016/j.carres.2007.02.033.
23. Frisch, M.J.; Trucks, G.W.; Schlegel, H.B.; Scuseria, G.E.; Robb, M.A.; Cheeseman, J.R.; ... Nakatsuji, H. GAUSSIAN09, Revision A.01. Wallingford, CT: Gaussian Inc., 2009.
24. Boys, S.F.; Bernardi, F.J.M.P. The calculation of small molecular interactions by the differences of separate total energies. Some procedures with reduced errors. *Mol. Phys*. **1970**, *19*, 553–566. https://doi.org/10.1080/00268977000101561.
